# Identification of *Thermus aquaticus* DNA polymerase variants with increased mismatch discrimination and reverse transcriptase activity from a smart enzyme mutant library

**DOI:** 10.1038/s41598-018-37233-y

**Published:** 2019-01-24

**Authors:** Govindan Raghunathan, Andreas Marx

**Affiliations:** Department of Chemistry, University of Konstanz, Universitätsstraße 10, D-78457 Konstanz, Germany

## Abstract

DNA polymerases the key enzymes for several biotechnological applications. Obviously, nature has not evolved these enzymes to be compatible with applications in biotechnology. Thus, engineering of a natural scaffold of DNA polymerases may lead to enzymes improved for several applications. Here, we investigated a two-step approach for the design and construction of a combinatorial library of mutants of KlenTaq DNA polymerase. First, we selected amino acid sites for saturation mutagenesis that interact with the primer/template strands or are evolutionarily conserved. From this library, we identified mutations that little interfere with DNA polymerase activity. Next, these functionally active mutants were combined randomly to construct a second library with enriched sequence diversity. We reasoned that the combination of mutants that have minuscule effect on enzyme activity and thermostability, will result in entities that have an increased mutation load but still retain activity. Besides activity and thermostability, we screened the library for entities with two distinct properties. Indeed, we identified two different KlenTaq DNA polymerase variants that either exhibit increased mismatch extension discrimination or increased reverse transcription PCR activity, respectively.

## Introduction

The invention of the polymerase chain reaction (PCR) is one of the most important innovations in biotechnology. Since the first report of its applications in diagnosis of sickle cell anaemia using *E. coli* DNA polymerase I^1,2^, this technique has progressed remarkably to different dimensions until now. The discovery of the thermostable *Thermus aquaticus* (Taq) DNA polymerase (a homolog of DNA polymerase I) and its ability to remain active after several rounds of repeated thermal cycling had expanded its applications to various fields of life science. Obviously, nature has not evolved the DNA polymerases to be compatible with applications in biotechnology. Thus, it turned out that engineering of wild-type DNA polymerases might lead to improved enzymes for several applications^3^. Strategies that were employed to deliver DNA polymerases with novel properties include site-directed mutagenesis based on rational design^4–6^, domain-tagging^7–10^, screening of DNA polymerase libraries by PCR or primer extension assays^11–13^, and *in vivo* or *in vitro* selection methods such as phage display^14,15^ and compartmentalized self replication (CSR)^16–20^.

In the past, we were succesful in obtaining DNA polymerase variants of KlenTaq DNA polymerase, the *N*-terminal shortened form of *Taq* DNA polymerase, that exhibit increased discrimination of matched vs. mismatched primer extension and demonstrate their capability in PCR applications like allele-specific amplification and genotyping^21^. We also identified KlenTaq DNA polymerase variants that have significant reverse transcriptase activity allowing direct reverse transcription combined with PCR from RNA targets^22^. These enzymes are applicable in reverse transcription PCR applications. The former enzymes that exhibit increased mismatch discrimination (or in other words: restricted substrate scope) were obtained from small focused libraries constructed by saturation mutagenesis at sites identified from structural data that are contacting the substrates. The later enzymes with the increased substrate scope (i.e., capability to use RNA as a template) were identified in libraries that were built from unfocused mutations on the entire enzyme scaffold through error-prone PCR. Interestingly, over the years, when searching for improved DNA polymerases, we failed to identify DNA polymerases with increased mismatch discrimination from unfocused libraries while we also failed to obtain DNA polymerases with reverse transcription activity from focused libraries.

Obviously, the usage of unfocused libraries suffers from the drawback of searching an astronomical number of sample sizes for new combinations of beneficial mutations, and also of the over-representation of non-functional protein sequences in the library. Therefore, rationalizing the amino acid position based on the knowledge of structural and evolutionary information, and generating a combinatorial library with functionally beneficial mutations would considerably improve the sequence diversity with increased functional protein sequences. Thus, we attempted to design a combinatorial library that coupled structure-based rational design and molecular shuffling of functional mutants. We reasoned that shuffling only active mutants for combinatorial library design would reduce the load of non-functional mutations in enzyme variants and thereby enrich the sequence diversity of the library with improved functional scope. Indeed, we identified two different KlenTaq DNA polymerase variants that either exhibit increased mismatch extension discrimination or increased reverse transcription PCR activity, respectively.

## Results

### General study design and selection of mutation sites

First, we aimed at constructing a focused library of KlenTaq DNA polymerase variants by rationally selecting target residues for mutation that were located in the proximity of the active site and make contact with primer/template DNA complex. Upon saturation mutagenesis at target residues and fluorescence-based screening, functional mutants should be identified. These mutants should be  shuffled in single reaction to construct a combinatorial library that was again screened for variants with improved mismatch discrimination and reverse transcription activity, respectively.

**Figure 1:**
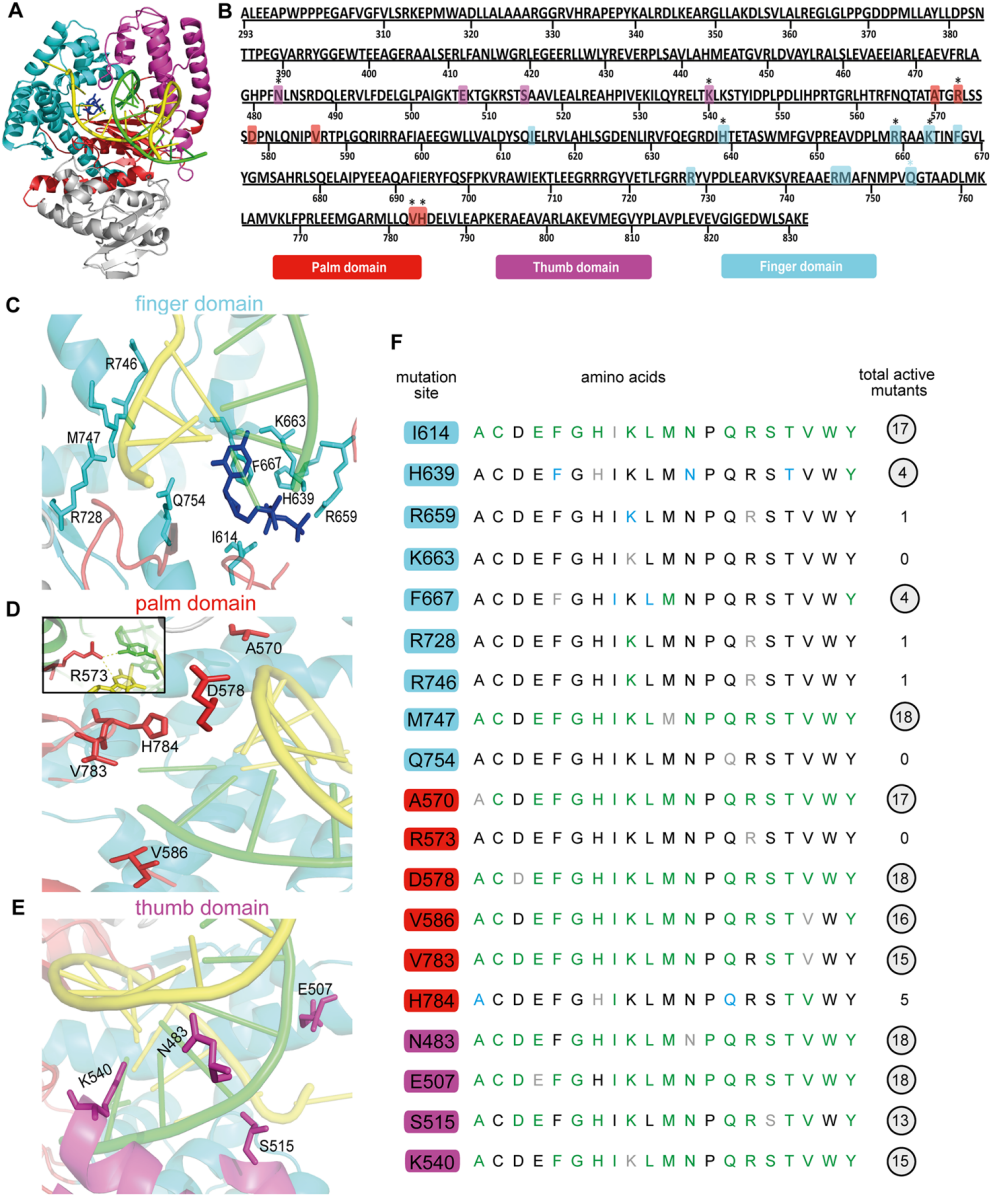
(**A**) Crystal structure of large fragment of DNA polymerase I (KlenTaq) from *Thermus aquaticus* (PDB ID 3KTQ)^23^. The N-terminal domain is highlighted in grey. The DNA complex containing primer (green) and template (yellow) is depicted. The finger, palm and thumb domains are depicted in cyan, red and magenta, respectively. (**B**) Rationally selected target sites for focused library construction are shown in the primary structure of KlenTaq DNA polymerase. The residues selected for mutations are highlighted in coloured boxes and the location of residues in different domains of KlenTaq DNA polymerase are shown in red (palm), magenta (thumb) and cyan (finger). Evolutionarily conserved residues are marked with asterisk sign on the top of amino acids. (**C**–**E**) Detailed view of rationally selected target residues from finger, palm and thumb domains, respectively. The incoming dideoxycytidine triphosphate is shown in dark blue. (**F**) Activity profile of target amino acid sites investigated by site-directed mutagenesis and denoted in their respective domain (finger, palm and thumb) colour code. Amino acid substitutions resulting in PCR active mutants are denoted in green (Cq 1–30) and the inactive mutants are shown in black (Cq >36). Blue indicates mutants with reduced activity (Cq 31–35) and the parent amino acids are highlighted in grey. Circled numbers indicate the active mutants included for molecular shuffling in the combinatorial library.

For the selection of sites to be mutagenized by saturation mutagenesis, we first inspected a crystal structure of a ternary complex composed of KlenTaq DNA polymerase (Fig. 1A,B)^23^. We reasoned that residues that make direct contacts with the primer/template DNA complex and incoming nucleoside-5′-*O*-triphosphate are promising candidates for mutagenesis, as these residues could significantly influence the substrate recognition and the catalytic property of KlenTaq DNA polymerase. These residues were selected for mutagenesis. In addition, we also focused on residues for mutation that are evolutionarily conserved in family A DNA polymerases from multiple sequence alignment of Taq, *E. coli* and T7 DNA polymerases, as mutations at such sites were shown to be promising to obtain altered functions^23–27^. The selected residues are mostly directed towards thumb, palm and finger domains since these regions form the primer/template DNA complex and nucleotide-binding crevice. We chose 19 residues as target sites for focused library generation: 9 residues from the finger domain, 6 residues from the palm domain and 4 residues from the thumb domain, respectively. A detailed view of the chosen sites which are either in proximity or in contact with primer/template DNA complex is depicted in Fig. 1C–E. Residues R728, R746, M747, Q754 (finger), A570, D578 (palm) and N483, (thumb) contact the template strand. Residues V586, (palm) and E507, S515, K540, (thumb) contact the primer strand. Residues I614, H639, F667, K663, R659 (finger) contact the incoming nucleoside-5′-*O*-triphosphate. Residues V783, H784 (palm) are highly conserved and located in proximity to primer strand. Residue R573 (palm) contacts both primer and template strand (Fig. 1D insert).

### Focused library: Construction and functional screening

After selecting the target sites for mutagenesis, we constructed the focused library of all possible 19 mutants for each target position. Each target site was mutated with oligonucleotides containing defined codons for the 19 individual amino acid exchanges using standard site-directed mutagenesis. The confirmed 361 mutants from the 19 target sites were established in 384 well plates and subsequently utilized for functional screening. Screening was performed with heat-denatured bacterial lysates as successful as before to identify active and inactive mutants using real time PCR^28^. For this purpose, a 92mer template DNA of the NANOG promoter region^29,30^ along with corresponding primers and SYBR^®^ Green I were employed in screening reaction and the data was recorded. The PCR active and inactive mutants were determined by measuring the respective Cq value, the cycle number at which the fluorescence signal crosses the background signal with an exponential increase of the signal. The screening results revealed that mutation at each position influenced activity to a varied extent (Fig. 1F). Of the positions investigated in palm and thumb domains, more than 60% and 70% mutants generated were found to be PCR active in each domain respectively (Fig. 1F). On the other hand, mutations in the finger domain resulted in an increased number of inactive mutants in comparison to palm and thumb domains, and only 25% PCR active mutants were found (Fig. 1F). The protein expression levels of selected mutants that are inactive were analysed by SDS-PAGE in order to elucidate that the reduction in activity is not due to reduced protein expression (see Supplementary Fig. S1).

### Generation of a combinatorial library with functional mutants

**Figure 2:**
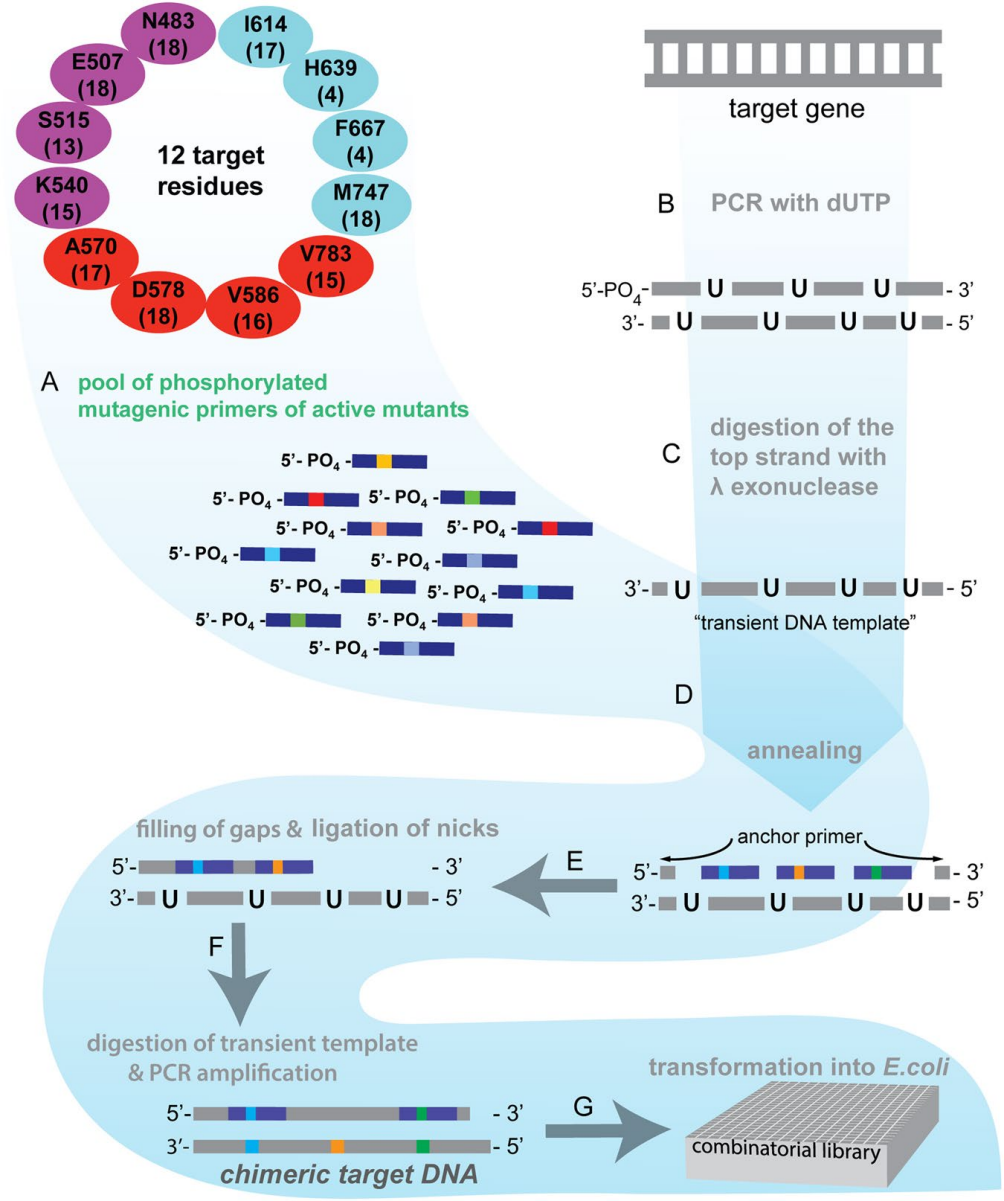
Scheme depicting the overall strategy for the construction of smart mutant library by molecular shuffling of active mutants. (**A**) Mutagenic oligonucleotides containing the codon for PCR active single mutants were pooled together and phosphorylated at 5′ end with T4 polynucleotide kinase. (**B**,**C**) A single strand transient DNA template of the target gene (KlenTaq DNA polymerase) was prepared by PCR in the presence of dUTP and subsequent digestion by λ exonuclease. (**D**,**E**) The mutagenic oligonucleotides were annealed and the gaps and nicks in the chimeric strand were filled and ligated. (**F**) The transient DNA template was cleaved and the remaining chimeric sequences were PCR amplified. (**G**) The recombinant plasmids were transformed into *E. coli* and grown on selection plate for the generation of combinatorial library.

Strategies involving degenerate oligonucleotides, gene fragments and synthetic DNA fragments were employed in the past to design combinatorial protein libraries^31–34^. Nevertheless, the rationale of shuffling several functional mutants of multiple target residues to construct a combinatorial library has not been attempted before. Here, we exploited the functional information of single site mutants achieved from the focused library to construct a combinatorial library for KlenTaq DNA polymerase by molecular shuffling (Fig. 2) and followed an approach that was described as RACHITT (random chimeragenesis on a transient template)^34^. We attempted to construct a combinatorial library with 173 active mutants from 12 target sites of KlenTaq DNA polymerase (Fig. 2). For this purpose, 173 mutagenic oligonucleotides were used, each containing defined mutations for the active mutant of focused library (see Supplementary Table S2). The mutagenic oligonucleotides of 173 active mutants were annealed with a transient single stranded template DNA of KlenTaq DNA polymerase that was obtained by PCR in the presence of dUTP instead of dTTP. The annealed mutagenic oligonucleotides 3′ ends were extended with Phusion U Hotstart DNA polymerase. After chimeric strand synthesis, the nicks were ligated with Taq DNA ligase and the transient single stranded template DNA was treated with uracil-DNA glycosylase to introduce abasic sites that foster strand cleavage under slightly basic conditions. Upon PCR amplification of chimeric strand and ligation into plasmid DNA, the recombinant plasmids were transformed into *E. coli* and grown on selection plate. Plasmids were prepared from randomly chosen recombinant clones and the sequences were analysed for the presence of chimerism at the defined mutational sites of KlenTaq DNA polymerase. The chimerism of mutants from the resulted library was verified by sequencing 20 random clones and the analysis of nucleotide sequences revealed that 85% of random clones had the mutations at least at one or two target sites (see Supplementary Fig. S2). Noteworthy, combinatorial library design involving shuffling of several numbers of functional mutants in position-specific manner, including mutagenesis of multiple target residues (12 target sites), has not been reported before^35^.

### Establishing and screening of combinatorial library for PCR activity

**Figure 3:**
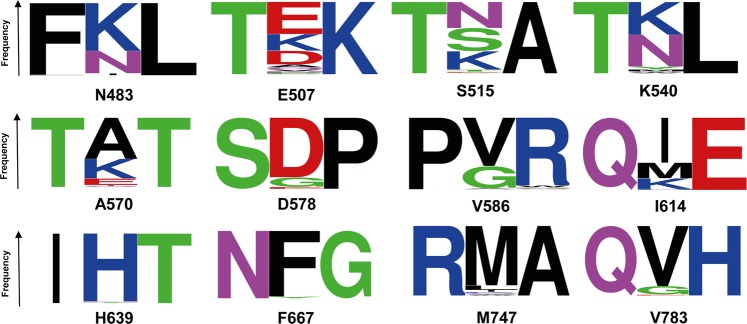
Sequence logo of PCR active mutant DNA polymerases (74 mutants), showing the frequency of amino acid exchange at 12 target sites of shuffled combinatorial library. For clarity, only the mutational target sites, flanked by their neighbouring amino acids of parent sequence, were shown in the figure^64^.

In order to generate the combinatorial library for activity screening, single cell clones were picked from the selection plate and cultivated in multi-well plates. A total of 15,000 recombinant clones were established. Overexpression was conducted in 96 well plate formats, and lysates were prepared for screening of functional mutants (PCR activity) as described above. The screen was performed using the corresponding template and primers, and enzyme activity recorded by quantifying the synthesised double stranded DNA using SYBR® Green I. About ~2000 mutants were identified to be PCR active with Cq value as high as <36 cycles to account for enzyme variants with impeded expression. The PCR active mutants were divided into four groups based on their Cq values. About 56% of mutants were active with Cq values of <10 cycles (Group_I), 27% were with Cq values between 10.1 to 20 cycles (Group_II), 11% were with Cq values between 20.1 to 30 cycles (Group_III) and 6% were with Cq values between 30.1 to 36 cycles (Group_IV). Seventy-four active mutants from the combinatorial library falling under the Group I and II category were sequenced. The sequence analysis revealed the amino acid substitutions in all active mutants at the target sites that were included in combinatorial library design. The protein sequence alignment of the active mutants showed the distribution of mutations and the frequency of amino acids exchange at each target site (Fig. 3).

### Mutant with improved discrimination between matched and mismatched primer/template substrates

**Figure 4:**
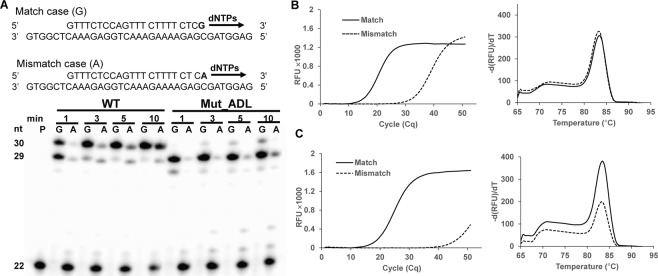
KlenTaq DNA polymerase mutant with improved allelic discrimination. (**A**) Sequence of employed primer-template pair. The 3′ end of primer either terminates with a matched (G) or mismatched (A) nucleotide. Primer extension reaction, employing the indicated DNA substrates along with wild-type (WT) and mutant DNA polymerase Mut_ADL, respectively, are shown as indicated. Reactions were stopped at different time points, as indicated. Full-length gel is presented in Supplementary Fig. S13 (**B**,**C**) Real time PCR experiments with purified enzymes (100 nM), showing the discrimination property of (**B**) wild-type KlenTaq DNA polymerase and (**C**) mutant DNA polymerase Mut_ADL respectively.

**Figure 5:**
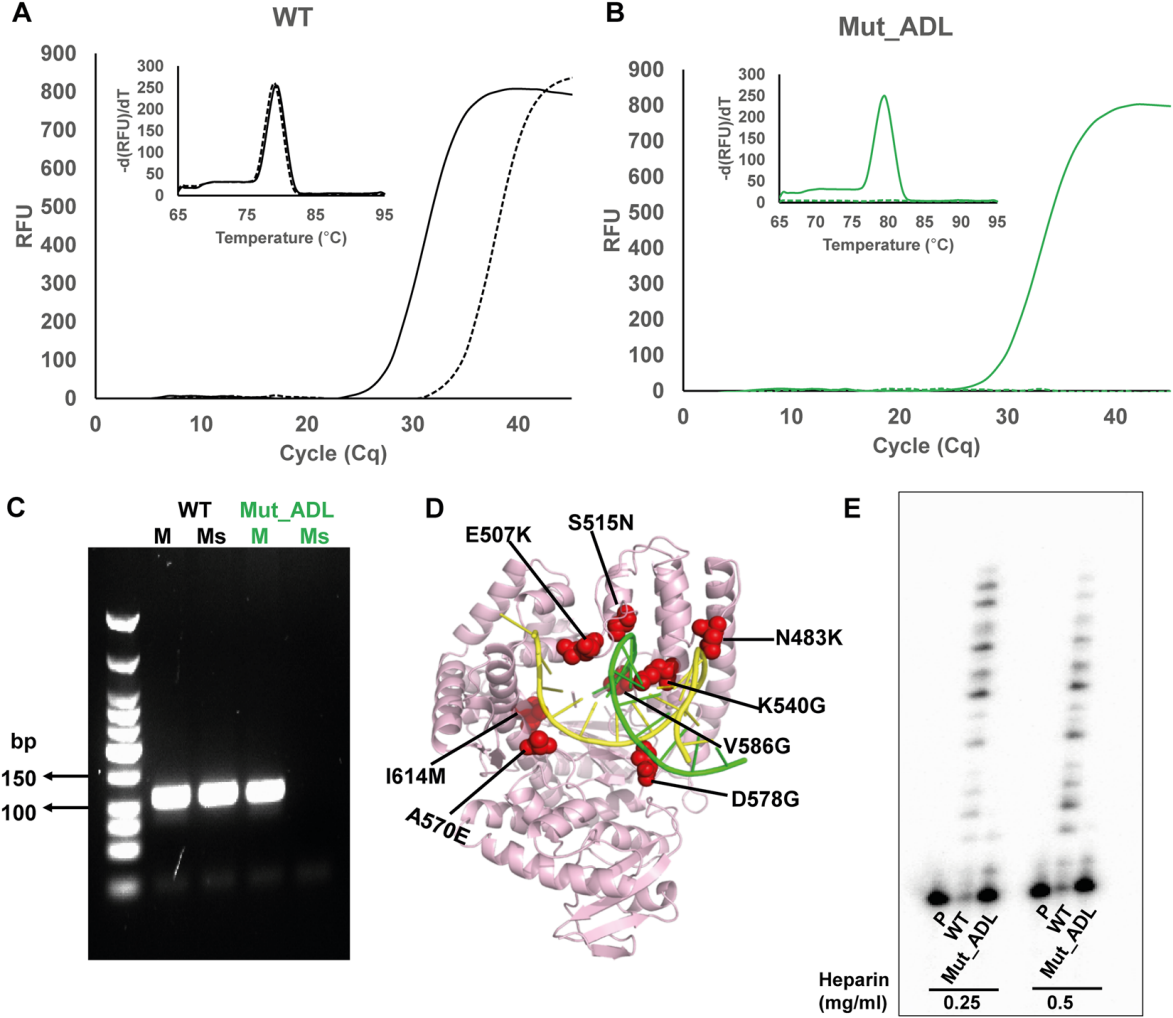
Allelic discrimination on genomic DNA context. Real time PCR experiments for the detection of single nucleotide variants within the olfactory receptor for wild-type KlenTaq DNA polymerase (WT) (**A**) and Mut_ADL (**B**) with HeLa genomic DNA. Match (solid) and mismatch (dashed) reactions shown in amplification plot. Inset graph, showing melting peaks for the products of respective PCR reactions of wild-type KlenTaq DNA polymerase and Mut_ADL. (**C**) Agarose gel electrophoresis, showing the PCR product (109 bp) of match (M) and mismatch (Ms) reactions of wild-type KlenTaq DNA polymerase and Mut_ADL, respectively. Full-length gel is presented in Supplementary Fig. S14. (**D**) Mutations contributing to improved discrimination property are depicted in crystal structure of KlenTaq DNA polymerase (in red). (**E**) Gel showing the processivity of wild-type KlenTaq DNA polymerase (WT) and Mut_ADL performed in presence of heparin (0.25 mg/ml and 0.5 mg/ml). Full-length gel is presented in Supplementary Fig. S15.

KlenTaq DNA polymerase mutants with enhanced discrimination between extending from matched and mismatched primer ends have potential in applications like allele specific amplification (ASA)^21,36,37^. Thus, we set up a screen to monitor allelic discrimination through real time PCR (qPCR)^38^. Two parallel PCR reactions; one with matched primer/template complex and the other with primer/template complex that has a single mismatch at the 3′-primer terminus, were conducted directly, using heat-denatured bacterial lysates containing mutant DNA polymerase. The amplification efficiency of both reactions was recorded in qPCR and the Cq determined respectively. The ∆Cq i.e., the difference in Cq value between both parallelly conducted reactions, is a measure for the allelic discrimination of the respective enzyme. Mutants showing higher ∆Cq than wild-type KlenTaq DNA polymerase were considered positive hits. In order to investigate the influence of protein expression and other factors, e.g., originating from components in lysate in the real time PCR reaction, selected positive hits were purified to homogeneity (see Supplementary Fig. S3) and investigated in primer extension experiments (see Supplementary Fig. S4). The best performing mutant, henceforth termed Mut_ADL, showed promising results in both, primer extension and real time PCR (Fig. 4). In primer extension, the Mut_ADL showed a significant difference in the propensity of extending the mismatch primer when compared with wild-type KlenTaq DNA polymerase (Fig. 4A). Wild-type KlenTaq DNA polymerase yielded 30 nt long reaction products due to non-templated addition of one extra nucleotide to the blunt DNA duplex, a phenomenon that was described for 3′-5′ exonuclease deficient DNA polymerases before^39,40^. Interestingly, the non-template reaction was reduced in case of mutant DNA polymerase. In qPCR experiments, the purified mutant enzyme exhibited a ∆Cq value of 26 cycles and on the contrary, wild-type KlenTaq DNA polymerase exhibited only 17 cycles. However, the mutant enzyme is somewhat less efficient in amplification from matched primer strand than the wild-type enzyme. Thus, the higher ∆Cq value of mutant enzyme confirmed the improved discrimination property of Mut_ADL. Melting-curve analysis revealed the amplification of a specific single PCR product during PCR reaction (Fig. 4B,C). Their corresponding melting peaks (right panels) confirmed the PCR products that were amplified by both DNA polymerases. Nucleotide sequence analysis of Mut_ADL revealed that the enzyme bears mutations at eight positions (see Fig. 5D and Supplementary Fig. S5). Noteworthy, the mutant DNA polymerase tolerated a mutation load of up to 8 amino acid substitutions that are involved in contacts with the primer/template complex. Next, to demonstrate the potential application of Mut_ADL’s increased discrimination, we investigated the enzyme activity regarding its property to detect single nucleotide polymorphism in genomic DNA. We thus chose the genomic SNP of olfactory receptor sequence context^41^ within HeLa genomic DNA. The increased propensity of Mut_ADL in comparison with the wild-type enzyme for allelic discrimination becomes more evident, as the mutant amplifies from a matched primer/template complex significantly more efficiently than from the mismatched (Fig. 5A–C).

In order to gain first preliminary insights into the overall selectivity of Mut_ADL, we also sequenced the amplificates of the PCR and found, that the sequence was maintained without any errors (see Supplementary Fig. S6). Additionally, we also investigated incorporation selectivity of matched versus mismatched nucleotides. The data obtained (see Supplementary Fig. S7) indicate no significant changes compared to the wild-type enzyme. Thus, the obvious increase in mismatch extension discrimination is not accompanied by an increase in insertion selectivity. This is not surprising since our screening design was set to focus on the former.

Next, we investigated the processivity of Mut_ADL in comparison to wild-type enzyme. We followed published protocols^42–44^ and used heparin as a trap. In brief, the primer template complex was preincubated with the enzyme prior to simultaneous addition of dNTPs and heparin. After incubation, the reactions were analysed by PAGE. Interestingly, while the wild-type extends the primer by incorporation of up to 13 nucleotides, the Mut_ADL is much more distributive and hardly starts synthesis and already aborts extension mostly after incorporation of one nucleotide (Fig. 5E). This distributive behaviour might add to the overall propensity of the enzyme to discriminate mismatches at the primer end.

In order to gain first insights whether the discrimination of mismatch extension also applies to other sequence contexts, we investigated Mut_ADL along these lines (see Supplementary Fig. S8). Much to our delight, the property of mismatch discrimination can also be found in the other sequence context. In summary, a viable mutant with potential application in allele-specific amplification that is used e.g., for genotyping, mutation diagnostics, and HLA typing^45–47^ with a hitherto unprecedented mutation load for a DNA polymerase with increased selectivity was discovered from the depicted combinatorial library design.

### Mutants with new catalytic activity: reverse-transcription PCR

**Figure 6:**
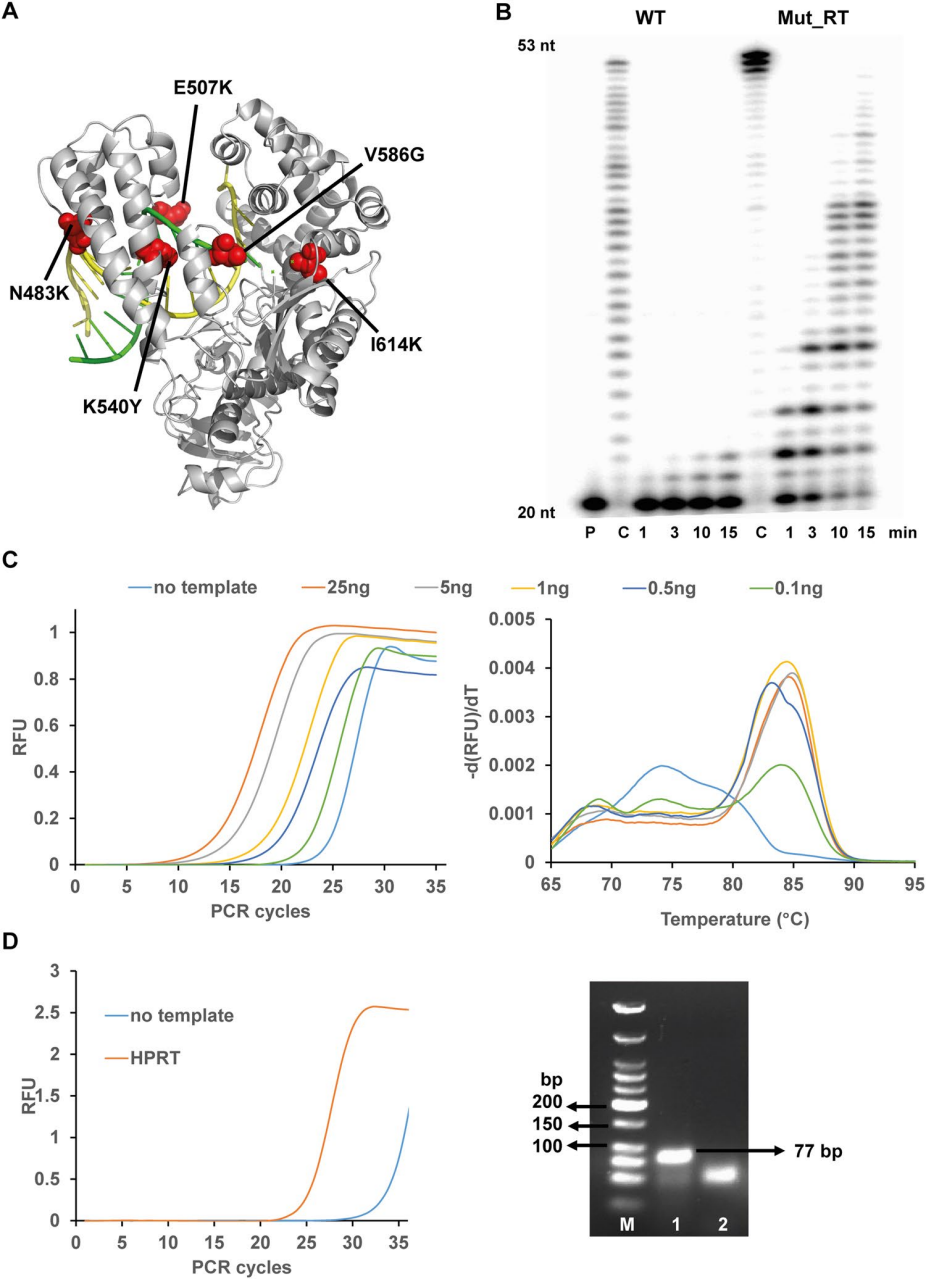
Evolved KlenTaq DNA polymerase with reverse transcriptase activity. (**A**) Depiction of novel mutations contributing to reverse transcriptase activity marked in red. (**B**) Reverse transcriptase activity studied by primer extension experiments for WT and Mut_RT. Reactions carried out for the indicated time points under identical conditions. P = primer, C = Control reaction that was carried out with the corresponding DNA template. Full-length gel is presented in Supplementary Fig. S16. (**C**) Reverse transcriptase activity of Mut_RT in real time PCR assay employed with varying amounts of RNA template (left) and their corresponding melting peak analysis (right). (**D**) Employability of Mut_RT in reverse transcription from total RNA extract (100 ng) using the HPRT mRNA target (left) and the agarose gel analysis of amplified product of HRPT transcript (lane 1) and the no template control (lane2) Full-length gel is presented in Supplementary Fig. S17.

The sequence and functional diversity of the constructed library was screened next for the identification of a functional property in which the wild-type enzyme shows little activity, namely the ability to reverse transcribe RNA into DNA^48,49^. The screen for reverse transcription PCR activity was again conducted from lysates of cells, expressing the mutated enzymes^22,50,51^. The enzymes were screened for reverse transcriptase (RT) activity through real time PCR, using MS2 bacteriophage genomic RNA as substrate for DNA synthesis. Mutants with RT activities were selected by quantifying PCR product formation with SYBR® Green I and by melting curve analysis in 96 well plate format. After screening the PCR active mutants of the combinatorial library, we identified several mutants with significantly increased RT activity. Afterwards, the RT active mutants were screened in a second round with more stringent conditions, involving reduced extension time of 7.5 min in initial reverse transcription step and reduced template concentration of 5 pg/ul. The most promising hit from this round (hitherto named Mut_RT) was sequenced and further characterized (Fig. 6). In Fig. 6A, the five mutations, conferring the novel polymerase activity, were depicted as well as the change in nucleotide sequence of the mutation sites in Supplementary Fig. S9. The new mutations showcased the functional plasticity of KlenTaq DNA polymerase and, more importantly, the sequence diversity contributing to activity on RNA template. The mutant was purified by Ni-NTA affinity chromatography and analysed on SDS-PAGE along with wild-type KlenTaq DNA polymerase (Supplementary Fig. S10). We characterized the mutant Mut_RT in detail, in comparison to wild-type KlenTaq DNA polymerase, by conducting primer extension and real time PCR experiments (Fig. 6). A radioactive labelled DNA primer strand was annealed to its complementary site on either 53 nt RNA template strand or DNA template strand and incubated with respective enzymes, wild-type KlenTaq DNA polymerase and Mut_RT. The extended products of primer extension experiments were resolved by 12% denaturing polyacrylamide gel electrophoresis. Both, wild-type and mutant were able to extend the primer strand to a full length product when the DNA template was used. However, with RNA template, it was evident that there was little intrinsic property of the wild-type polymerase to utilize an RNA template, as it failed to extend the bound DNA primer strand beyond two nucleotides (Fig. 6B). On the other hand, Mut_RT showed remarkable extension of the primer strand when hybridized with an RNA template (Fig. 6B). The specific activity using RNA and DNA templates was investigated in comparison to the wild-type enzyme (Table 1, Supplementary Fig. S11). This confirmed the superiority in reverse transcription of the identified mutant, compared to the parental enzyme. Interestingly, the activity of Mut_RT on DNA template was 4-fold higher in comparison to wild-type KlenTaq DNA polymerase. This effect was observed for other DNA polymerases such as other variants of KlenTaq DNA pol and human DNA polymerase β that were evolved to have increased reverse transcriptase or lesion bypass activity^22,50,52,53^.

**Table 1:** Specific activities of KlenTaq DNA polymerase variants on DNA or RNA template.

	Specific activity × 10^2^ [min^−1^] DNA template	Specific activity × 10^2^ [min^−1^] RNA template
Wild-type	6.58 ± 0.36	n.d.
Mut_RT	28.87 ± 0.96	1.94 ± 0.1

Next, the reverse transcriptase activity of the evolved enzyme was investigated by employing varied concentration of RNA template in qPCR with SYBR^®^ Green I for detection of double stranded DNA (Fig. 6C). The sensitivity of Mut_RT in the employed qPCR was about 0.1 ng, whereas the wild-type enzyme showed no amplification under identical conditions. Finally, to demonstrate the applicability of Mut_RT in cDNA synthesis, total RNA from HEK293 cells was extracted and tested as target for Mut_RT for the amplification from the target HRPT mRNA^54^. The desired 77 bp PCR product was amplified from the HRPT mRNA transcript as detected in agarose gel electrophoresis (Fig. 6D).

Again, in order to gain first preliminary insights into the overall selectivity of Mut_RT, we also sequenced the amplificates of the PCR and found, that the sequence was maintained without any errors (see Supplementary Fig. S12). The above findings show that the identification of a catalytic function that is not present in the parental enzyme could be achieved by the employed combinatorial library design. We identified a new set of mutations that result in reverse transcription activity. We expect that the novel mutations of Mut_RT can serve as an evolutionary starting point for further engineering studies^12,55^.

## Discussion

Here, we investigated a two-step approach that coupled structure-based design and molecular shuffling for the design and construction of a combinatorial library of mutants of KlenTaq DNA polymerase. First, we selected target amino acid sites for saturation mutagenesis that are making direct interaction with the primer/template strands or evolutionarily conserved among family A DNA polymerases. The rationally designed library was investigated extensively for the effect of amino acid substitution on each target site, and the activity profile showed some of the selected target sites to remain immutable (Fig. 1F). For instance, Arginine 573 directly interacts with the nucleobase of both, primer and template strands and could not tolerate any mutational substitution, thereby suggesting its crucial role in structural stability and catalytic function of DNA polymerase (Fig. 1D Inset). Residues R659, K663, R728, R746 and Q754 of finger domain were also least tolerant to the substitution (Fig. 1F). Overall, from this library we identified mutations that had negligible interference with DNA polymerase activity and thermostability. Then these functionally active mutants were randomly combined to build a versatile second library. We reasoned that the combination of mutants that have minuscule effect on enzyme activity and thermostability would result in entities that have an increased mutation load, but still retain activity. Besides activity and thermostability, we screened the library for entities with two distinct properties.

First, we screened the library for entities with increased mismatch discrimination properties when the mismatches were located at the 3′-primer terminus. We identified the variant Mut_ADL that significantly discriminates between mismatches at the terminus and has high potential for applications like allele-specific amplification or genotyping (Fig. 5). Interestingly, the mutant Mut_ADL bears eight mutations contrasting our earlier attempts along the lines where only competent variants, bearing single mutants, were identified and further mutation results in significantly inactivated enzymes^21^. We also found that four of the eight mutations are located in evolutionarily conserved motifs of family A DNA polymerase (N483K in Motif 1, A570E, D578G in Motif 2, & I614M in Motif A)^24,56^. We speculate that such a tremendous tolerance of mutation load was nearly feasible due to the rational of combining functionally active mutants for the combinatorial library design.

Next, we screened the library for entities that exhibit reverse transcription activity, a catalytic activity that is only minuscule in the parental enzyme. Again, we were successful in identifying a variant. The variant termed Mut_RT bears five mutations and exhibits significantly increased activity on RNA as well as DNA, in comparison to the wild-type enzyme. The mutations of Mut_RT DNA polymerase are novel to our knowledge (Fig. 6A). It has been known that the mutation E507K in Taq DNA polymerase contributes to a fast PCR cycling property^57^, and mutation at K540, in concert with five other mutations, displays increased resistance to inhibitors, such as heparin^58^. Residue I614 tolerates amino acid exchange and retains activity near to the wild-type enzyme, but confers to low fidelity^59^. I614M in combination with secondary mutations (E602V, A608V, E615G) exert the functions of synthesising DNA-RNA hybrid^60^. However, the single mutation I614K has been shown to incorporate both deoxyribonucleotides and ribonucleotides with DNA template but not with RNA template^61^. From this, we speculate that the reverse transcriptase activity of Mut_RT might be due to the synergistic effects of distal mutations (N483K, E507K, I614K, K540Y, V586G).

The obtained results support our hypothesis that the depicted approach – rational design of mutation sites and combinatorial design by shuffling active mutants – is indeed successful in identifying enzyme variants with a diverse functional scope. We were successful in obtaining an enzyme with limited substrate scope (extension from matched primer strands) and with increased substrate scope (efficient usage of RNA templates). This is the first time that we were able to “evolve” these seemingly incompatible properties from a single library. We envision that this method is not only limited to the functions as described herein. Future attempts to explore the impact of sequence diversity on enzyme function of this library will aim to screen for mutants e.g., to sense epigenetic marks at single base resolution, the propensity to process damaged templates, improved propensity for chemically modified substrates, and improved tolerance to inhibitors. Of note, we reason, that the approach depicted here is not limited to KlenTaq DNA polymerase but can also be extended to other proteins or enzymes where structural data is readily available.

## Material and Methods

### DNA manipulation and focused library construction

We purchased oligonucleotides from biomers.net GmbH and dissolved them in deionized water. The single mutants for all the selected target sites were generated by site-directed mutagenesis. Forward primers (Mutagenic primers) were designed to carry the triplet codon for the desired mutation and 5′ phosphorylated reverse primers aided in ligation after PCR. Each target residue was mutated with 19 different forward primers in a PCR reaction containing Phusion^®^ High fidelity DNA polymerase (NEB) and other necessary PCR components. After PCR amplification, the template plasmid (wild-type KlenTaq in pGDR11) was digested using the methylation sensitive endonuclease DpnI (NEB) by incubating at 37 °C for 1 hr. The PCR products were purified from agarose gel with QIAquick^®^ Gel Extraction Kit (QIAGEN) and ligated with T4 DNA ligase (NEB) overnight in the refrigerator. The ligated products were transformed into calcium chloride treated *Escherichia coli* BL21 (DE3) cells (Novagen) and positive clones were selected after overnight incubation at 37 °C. Plasmids were extracted from positive clones using QIAprep^®^ Spin Miniprep Kit (Qiagen) and sequenced by Sanger sequencing (GATC Biotech). Clones carrying the desired mutations were established in 384 well plate containing 150 μl of LB media amended with 100 μg/ml of carbenicillin and incubated for overnight growth. The overnight grown cultures were stored at −80 °C after adding 50 μl of 60% sterile glycerol (v/v).

### Overexpression of KlenTaq DNA polymerase library

Overexpression of KlenTaq DNA polymerase mutant library was carried out as already described^28^. In short, the expression was performed in 96 well plates with 10 μl of overnight grown culture in 940 μl of LB media containing 100 μg/ml of carbenicillin. Protein expression was induced with 1 mM IPTG and cells were harvested by centrifugation at 4000 rpm for 20 min. Cell pellets were resuspended in 1X KlenTaq buffer (50 mM Trizma^®^ base (pH 9.2), 16 mM (NH_4_)_2_SO_4_, 2.5 mM MgCl_2_, 0.1% (v/v) Tween20) containing 0.1 mg/ml lysozyme and lysed at 37 °C water bath for 20 min. After heat denaturation at 75 °C for 45 min, the plates were centrifuged at 4000 rpm at 4 °C for lysate preparation and the lysates were used directly for screening.

### Screening for functional KlenTaq DNA polymerase mutants

Typically, activity screening was performed in 10 μl reaction volume containing 1X KlenTaq reaction buffer (50 mM Trizma^®^ base (pH 9.2), 16 mM (NH_4_)_2_SO_4_, 2.5 mM MgCl_2_, 0.1% (v/v) Tween20), 200 μM dNTPs, 50 nM forward primer (5′-d(CTT GGT GAG ACT GGT AGA CG)-3′), 50 nM reverse primer (5′-d(TTA GAC CCA CCC CTC CTG GCG)-3′), 100 pM DNA template (sequence in Supplementary Table S1), 1X SYBR^®^ Green I (Sigma). The data output for PCR activity was collected in Biorad CFX 384^TM^ real time PCR with the following cycling conditions: Initial denaturation at 95 °C for 1 min, 50 PCR cycles of 95 °C for 10 s, 65 °C for 10 s and 72 °C for 10 s. The data was analysed using Bio-Rad CFX Manager 3.1. The selected active mutants were used further in combinatorial design through shuffling.

### Construction of Combinatorial library

Oligonucleotides (Mutagenic primers) of selected active mutants were mixed in one pot for 5′ end labelling (Supplementary Table S2). Reactions containing 200 pmol of mutagenic primers mixture, 1X T4 DNA ligase buffer (NEB), 10 U of T4 polynucleotide kinase were incubated at 37 °C for 60 min and heat inactivated at 75 °C for 10 min. A transient single stranded DNA scaffold was used as template in molecular shuffling and prepared as described in the previously published protocol^34,62^. The shuffling of active mutants involved four steps: annealing, gap filling/ligation, uracil-DNA glycosylase treatment and PCR of chimeric DNA. Phosphorylated mutagenic primers (20 pmol) along with transient ssDNA template (25 fmol) in presence of two other oligonucleotides [(5′-d(TCT GGC AGG CCA TCC GTT T)) & (5′-P-d(AAG CTT AAT TAG CTG AGC TTG GAC TCC TGT)] for 5′ and 3′ termini (1.3 pmol each) were annealed in 7.5 μl reaction volume, containing 1 μl of 10X Taq DNA ligase buffer (NEB). Annealing was done in Biometra thermocycler with the thermal conditions; 95 °C for 2 min, 75 °C for 3 min and then gradual cooling to 50 °C over 40 min (at the ramp rate of −0.6 °C/min) and finally held on ice for 10 min. The gaps between the successively annealed mutagenic primers were filled and ligated in a separate reaction (20 μl), containing 7.5 μl of annealed reaction mix, 1 μl of 10X Taq DNA ligase buffer, 1 μl of 10 mM dNTPs, 1 U of Phusion U Hotstart DNA polymerase (Thermo Fischer Scientific) and 32 U of Taq DNA ligase (NEB). The reaction was started at 45 °C and ramped to 65 °C over 30 min (with ramp rate of 0.6 °C/min). Then the reaction mixture was incubated for 90 min at 65 °C. The transient ssDNA template was digested with uracil-DNA glycosylase (UDG) at 37 °C for 40 min in 10 μl reaction, containing 1X UDG buffer, 1 mg/μl BSA and 5 U of UDG. The chimeric molecules were PCR amplified with two primers, containing FseI (5′-d(GAA GTT TTT CGT CTG GCC GGC CAT) and HindIII (5′-d(ACA GGA GTC CAA GCT CAG CTA ATT AAG CTT) and purified from agarose gel using QIAquick^®^ Gel Extraction Kit (QIAGEN). After restriction digestion with HindIII and FseI at 37 °C and heat inactivation at 65 °C for 20 min, the products were purified by QIAquick PCR Purification Kit and ligated into pGDR11 vector harbouring similar restriction sites overhangs. The ligated products were transformed into the chemical competent host NEB Express Competent *E. coli* (High efficiency). Random recombinant clones were picked for plasmid extraction, using QIAprep^®^ Spin Miniprep Kit (Qiagen) and analyzed for the presence of mutations by Sanger sequencing (GATC Biotech). The recombinant clones were picked, established and cultured in 384 well plates for PCR activity screening. PCR active mutants were screened with real time PCR as described previously and a library of KlenTaq DNA polymerase active mutants were established and stored at −80 °C. Handling was done in multi-channel pipettes and manual high-throughput Liquidator™ 96-channel benchtop pipettor.

### Expression and purification of KlenTaq DNA polymerase wild-type and variants

For homogenous protein preparation, the wild-type and mutant polymerases were grown in either 100 or 200 ml of LB media containing 100 μg/ml carbenicillin at 37 °C^52^. After OD_600nm_ reaches 0.6–0.8, the protein expression was induced with 1 mM IPTG and overexpressed for 4–5 hrs. The cells were harvested by centrifugation at 4000 rpm at 4 °C for 20 min and stored at −20 °C for further use. Cell pellets were resuspended in 1X KlenTaq buffer, containing 0.1 mg/ml lysozyme and incubated at 37 °C for 20 min. After heat denaturation at 75 °C for 45 min in water bath, the lysates were centrifuged at 20,000 × g and 4 °C for 45 min. The supernatant was filtered through a 0.2 μm syringe filter and mixed with 50% nickel beads slurry (Roche) for 90 min incubation at 4 °C. After incubation, the beads were washed with wash buffer, containing 10 mM Trizma^®^ base (pH 9.0), 2.5 mM MgCl_2_, 300 nM NaCl and 15 mM imidazole. The proteins were eluted with elution buffer (100 mM Trizma^®^ base (pH 9.2), 5 mM MgCl_2_, 100 mM imidazole). The excess imidazole in elution fraction was removed through ultrafiltration unit (Vivaspin 20, (30,000 MWCO PES)). Proteins were stored at −20 °C in buffer, containing 50 mM Trizma^®^ base (pH 9.2), 2.5 mM MgCl_2_, 16 mM (NH_4_)_2_SO_4_, 0.1% Tween 20, 50% glycerol. The concentration of purified proteins was determined by Bradford’s reagent.

### 5′ phosphorylation of oligonucleotides using radioactive ATP

Oligonucleotides for primer extension experiments were radioactively labelled at 5′ end using [γ-^32^P]-ATP and T4 polynucleotide kinase (PNK). Reactions (50 μl) containing 1X T4 PNK buffer, 0.4 μM of oligonucleotides, 0.4 U/μl T4 PNK and 0.4 μC/μl [γ-^32^P]-ATP were incubated at 37 °C for 60 min and stopped by incubating at 95 °C for 2 min. After removal of excess ATP and additional salts through gel filtration by Sephadex G-25 microspin column (GE Healthcare), 20 μl of unlabelled primer (10 μM) was added to get a final concentration of 3 μM diluted radioactive labelled primer for use in primer extension experiments.

### Allelic discrimination assay (with lysate or purified enzyme) and primer extension assay

Reaction (10 μl) contained 1X KlenTaq reaction buffer, 200 μM dNTPs and 0.6X SYBR^®^ Green I (Sigma). As template, 92 nt 100 pM DNA template (sequence in Supplementary Table S1) was used with 50 nM primer either M_For [5′ -d(CTT GGT GAG ACT GGT AGA C**G**) 3′] or Ms_For [5′ -d(CTT GGT GAG ACT GGT AGA C**A**) 3′] and 50 nM Rev_primer [5′-d(TTA GAC CCA CCC CTC CTG GCG) 3′] in two separate reactions. Both primers M_For and Ms_For contained the SNP at 3′ terminus. After an initial denaturation at 95 °C for 1 min, the thermal cycling was repeated for 50 cycles (95 °C for 10 s, 65 °C for 10 s and 72 °C for 10 s) and the reaction was analysed by real-time PCR curves and melting curve measurement within the cycler Biorad CFX 384^TM^. Lysates were prepared, as described before, and directly used for screening allelic discrimination assay. All reactions with purified enzymes were repeated and performed at least thrice. For primer extension assay, the reaction mixture (20 μl) contained 1X KlenTaq reaction buffer, 250 nM template (5′-d(GAG GTA GCG AGA AAA GAA ACT GGA GAA ACT CGG TG)3′), 150 nM of 5′radioactively labelled primers (either Match primer or Mismatch primer, carrying SNP at 3′ end), 20 nM purified enzyme. After pre-incubation at 95 °C for 2 min, the reaction was started at 55 °C by the addition of 100 μM dNTPs and terminated at different time interval with 2 volumes of stopping solution (80% [v/v] formamide, 20 mM EDTA, 0.25% [w/v] bromophenol blue, 0.25% [w/v] xylene cyanol). Products were resolved in 12% denaturing PAGE and visualized using Phosphorimager. The images were prepared with the Quantity One software from Biorad.

### Single nucleotide variants detection assay with genomic DNA

Reaction mixtures (10 μL) contained 1X KlenTaq reaction buffer, 200 μM of each dNTP, 0.2X SYBR^®^ Green I, 1 ng/μl HeLa genomic DNA, 100 nM Taq DNA polymerase aptamer [5′-d(CGA TCA TCT CAG AAC ATT CTT AGC GTT TTG TTC TTG TGT ATG ATC G)3′]^63^ and 100 nM of the respective DNA polymerase. As a forward primer, either match primer [200 nM, 5′-d(GAA TGG GAT AGA GAA GGG ATC AAA AG)] or mismatch primer [200 nM, 5′-d(GAA TGG GAT AGA GAA GGG ATC AAA AT)] was used together with reverse primer [200 nM, 5′-d(CTG CTG CTT GAA AAT GGA TTG TG)]. Real-time PCR was performed with an initial denaturation cycle (95 °C for 3 min), followed by 45 PCR cycles (95 °C for 10 s, 57 °C for 15 s and 72 °C for 30 s) and analysis of an amplicon size of 109 bp by melting-curve analysis within the device (Biorad CFX 384^TM^).

### Processivity assay

Reaction mixture (20 μl) contained 1X KlenTaq reaction buffer, 40 nM template [5′-d(GAA CGG CCG GGC GCG GTG GCC CAC CCC TGT AAT C)3′], 40 nM of 5′ radioactively labelled primer [5′-d(ATT ACA GGG GTG GGC CAC CG)3′], 4 nM purified enzyme. After pre-incubation at 95 °C for 2 min and cooling to 55 °C, the reaction was started by the addition of 200 μM dNTPs and heparin (either 0.25 or 0.5 mg/ml). The reaction was run for 10 min and stopped with one volume of stopping solution (80% [v/v] formamide, 20 mM EDTA, 0.25% [w/v] bromophenol blue, 0.25% [w/v] xylene cyanol). The products were resolved in 12% denaturing polyacrylamide gel electrophoresis and visualized using Phosphorimager. The images were prepared with the Quantity One software from Biorad.

### Activity screening using RNA template (with lysate) and primer extension assay

Assay reaction (20 μl) contained 1X KlenTaq reaction buffer, 200 μM dNTPs, 100 nM RT_For (5′-d(ATC GCT CGA GAA CGC AAG TT)3′), 100 nM RT_Rev (5′-d(CG GAC TTC ATG CTG TCG GTG)3′), 50 pg/μl of MS2 bacteriophage genomic RNA template (Roche), 0.6X SYBR^®^ Green I (Sigma) and 5 μl lysate^22,50^. Real time PCR screening assay was performed with an initial reverse transcription step (initial denaturation at 95 °C for 30 s, annealing at 55 °C for 35 s and extension at 72 °C for 15 min) and 50 PCR cycles (95 °C for 30 s, 55 °C for 35 s and 72 °C for 40 s) in Light Cycler^®^ 96 instrument (Roche). A second screen, with 5 pg/μl MS2 RNA template and reduced extension time in initial reverse transcription step (Extension at 72 °C for 7.5 min), was employed for mutants selected from first screen. The primer extension assay reaction mixture (20 μl) contained 1X KlenTaq reaction buffer (50 mM Trizma^®^ base (pH 9.2), 16 mM (NH_4_)_2_SO_4_, 2.5 mM MgCl_2_, 0.1% (v/v) Tween20), 225 nM RNA template (5′ AUA GGG GAA UGG GCC GUU CAU CUG CUA AAA GGA CUG CUU UUG GGG CUU GUA GU 3′), 150 nM of [γ-^32^P]-labelled primer (5′-d(ACT ACA AGC CCC AAA AGC AG)3′) and 2 nM of purified respective enzymes. After pre-incubation at 95 °C for 2 min and cooling to 55 °C for 2 min, the reaction was started by adding 200 μM dNTPs at 55 °C and stopped at different time points by adding 40 μl stopping solution (80% [v/v] formamide, 20 mM EDTA, 0.25% [w/v] bromophenol blue, 0.25% [w/v] xylenecyanol). For control purposes, 5 nM of purified enzymes were employed with the corresponding DNA template and stopped after 1 min of incubation. Products were resolved in 12% denaturing PAGE and visualized using Phosphorimager. The image was prepared with the Quantity One software from Biorad.

### Specific activity of KlenTaq DNA polymerase variant

Primer extension assay was performed as described above with an incubation time of 1 min for either DNA or RNA as template. Various amounts of Mut_RT polymerase employed in reactions for DNA or RNA templates were 1, 2.5, 5, 10, 15, 20 fmol or 5, 10, 20, 40, 60, 80, 100 fmol, respectively. For wild-type KlenTaq DNA polymerase, the various amounts of polymerase used for DNA template were 6.25, 12.5, 25, 50, 75, 100 fmol. The reaction products were analysed by 12% denaturing PAGE and visualized via phosphorimaging. The observed intensities of each band yielded the conversion of dNTPs in each reaction. dNTP conversion per min was then plotted against the amount of enzymes. The linear range was analysed and slopes were obtained using linear regression (Supplementary Fig. S11) yielding the specific activity of the respective enzyme.

### Reverse transcription PCR with MS2 phage RNA template dilution series

Reaction (20 μl) contained 1X KlenTaq reaction buffer, 200 μM dNTPs, 100 nM RT_For (5′-d(ATC GCT CGA GAA CGC AAG TT)3′), 100 nM RT_Rev (5′-d(CG GAC TTC ATG CTG TCG GTG)3′), varied amount of MS2 bacteriophage genomic RNA template (Roche), 0.6X SYBR^®^ Green I (Sigma) and 50 nM of purified enzyme. Reverse transcription PCR was performed with an initial three steps (initial denaturation at 95 °C for 30 s, annealing at 55 °C for 35 s and extension at 72 °C for 7.5 min) and 35 cycles (95 °C for 30 s, 55 °C for 35 s and 72 °C for 40 s) in Light Cycler^®^ 96 instrument (Roche). The amplified products were analysed by melting curve measurement. Data was collected from triplicate reactions and analysed using Light Cycler^®^ 96 software.

### Reverse transcriptase activity using total cellular RNA extract

Total RNA extraction from HEK293 cells was carried out using Direct-zol™ RNA Miniprep kit (Zymo Research) and in-column DNA digestion was performed according to manufacturer’s protocol. RNA concentration was determined using NanoDrop^TM^ 1000 Spectrophotometer (PEQLAB). Reaction (20 μl) contained 1X KlenTaq reaction buffer (50 mM Trizma^®^ base (pH 9.2), 16 mM (NH_4_)_2_SO_4_, 2.5 mM MgCl_2_, 0.1% (v/v) Tween20), 200 μM dNTPs, 5 ng/μl total RNA extract as template, 1X SYBR^®^ Green I (Sigma), 50 nM purified enzyme, 200 nM forward primer (5′-d(ATG GGA GGC CAT CAC ATT GT)-3′) and 200 nM reverse primer (5′-d(ATG TAA TCC AGC AGG TCA GCA A)-3′) for HPRT transcript (77 bp). Amplification was carried out with initial steps of denaturation (95 °C for 1 min) and reverse transcription (60 °C for 7 min), and 45 cycles of two step protocol with 95 °C for 15 s and 65 °C for 30 s in Light Cycler^®^ 96 instrument (Roche).

## Supplementary information

Supplementary Information
