# Supplementary material for: Identification of Thermus aquaticus DNA polymerase variants with increased mismatch discrimination and reverse transcriptase activity from a smart enzyme mutant library
Source: Sci Rep. 2019 Jan 24;9:590. doi: 10.1038/s41598-018-37233-y (PMC6345897; doi:10.1038/s41598-018-37233-y)
Supplement: Supplementary file 1 — Supplementary Information [file 41598_2018_37233_MOESM1_ESM.pdf]

Identification of *Thermus aquaticus* DNA polymerase variants with increased mismatch discrimination and reverse transcriptase activity from a smart enzyme mutant library

Govindan Raghunathan and Andreas Marx*

Department of Chemistry, University of Konstanz, Universitätsstraße 10, D-78457 Konstanz, Germany

*Correspondence should be addressed to Email: andreas.marx@uni-konstanz.de

Supplementary Data

CONTENTS

Table S1. Oligonucleotide template used in this study

Table S2. Mutagenic oligonucleotides of active mutants

Figure S1. Analysis of expression profile of selected target sites by SDS-PAGE

Figure S2. Nucleotide sequence analysis of random mutants from the combinatorial library to confirm mutagenesis at multiple target sites

Figure S3. SDS-PAGE analysis of affinity purified KlenTaq DNA polymerase variants

Figure S4. Primer extension analysis of KlenTaq variants for increased mismatch discrimination

Figure S5. Nucleotide sequence analysis of KlenTaq variant Mut_ADL to depict the mutational sites

Figure S6. Sanger sequencing analysis of amplified products

Figure S7. Single nucleotide misinsertion assay for wild type KlenTaq DNA polymerase and Mut_ADL

Figure S8. Improved allelic discrimination on different sequence context

Figure S9. Sequence alignment of Mut_RT and wild type to reveal the change of codon at mutational sites

Figure S10. SDS-PAGE analysis of affinity purified Mut_RT along with wild type

Figure S11. Specific activities of KlenTaq DNA polymerase variants on DNA or RNA template

Figure S12. Sanger sequencing analysis of Mut_RT amplified products

Table S1. Oligonucleotide template used in this study

Label	Sequence
DNA template	5'-d(ACC TTA GAC CCA CCC CTC CTG GCG GGC ACA CCC CCT ACT GAC CCA CCC TTG TGA ATT CTC AGT TAA TCC CGT CTA CCA GTC TCA CCA AGG CC)3'
DNA template_1	5’-d(AGC AAC AAA TAC TTC TAG GTT CAC CAC GTT TCC AAC TTT TAA ATC AGG AAT ATG GTT CAA CAG GAA TGG GAT AAA ACT TTA ATA TTT TTA AAA ATT AAG AAA AAG GCC GGG CGC GGT GGC CCA CCC CTG TAA TCC CA) 3’

Table S2. Mutagenic oligonucleotides of active mutants

Table showing selected 12 target sites for site-specific shuffling to construct combinatorial library. Active single mutants of the respective target sites were included for shuffling and the Mutagenic oligonucleotide of 173 active mutants were pooled in shuffling reaction.

	N483	E507	S515	K540	A570	D578	V586	V783	I614	H639	F667	M747
Ala	✓	✓	✓	✓	+	✓	✓	✓	✓	×	×	✓
Cys	✓	✓	×	×	✓	✓	✓	✓	✓	×	×	✓
Asp	✓	✓	✓	×	×	+	×	✓	×	×	×	×
Glu	✓	+	✓	×	✓	✓	✓	✓	✓	×	×	✓
Phe	×	✓	×	✓	✓	✓	✓	✓	✓	✓	+	✓
Gly	✓	✓	✓	✓	✓	✓	✓	✓	✓	×	×	✓
His	✓	×	✓	✓	✓	✓	✓	✓	✓	+	×	✓
Ile	✓	✓	×	✓	✓	✓	✓	✓	+	×	✓	✓
Lys	✓	✓	✓	+	✓	✓	✓	✓	✓	×	×	✓
Leu	✓	✓	×	✓	✓	✓	✓	✓	✓	×	✓	✓
Met	✓	✓	✓	✓	✓	✓	✓	✓	✓	×	✓	+
Asn	+	✓	✓	✓	✓	✓	✓	✓	✓	✓	×	✓
Pro	✓	✓	×	×	×	×	×	×	×	×	×	✓
Gln	✓	✓	✓	✓	✓	✓	✓	✓	✓	×	×	✓
Arg	✓	✓	✓	✓	✓	✓	✓	×	✓	×	×	✓
Ser	✓	✓	+	✓	✓	✓	✓	✓	✓	×	×	✓
Thr	✓	✓	✓	✓	✓	✓	✓	✓	✓	✓	×	✓
Val	✓	✓	✓	✓	✓	✓	+	+	✓	×	×	✓
Trp	✓	✓	×	✓	✓	✓	×	×	✓	×	×	✓
Tyr	✓	✓	✓	✓	✓	✓	✓	×	✓	✓	✓	✓

'✓' Active mutant included in combinatorial library construction
'+' Wild type
'×' Inactive variant

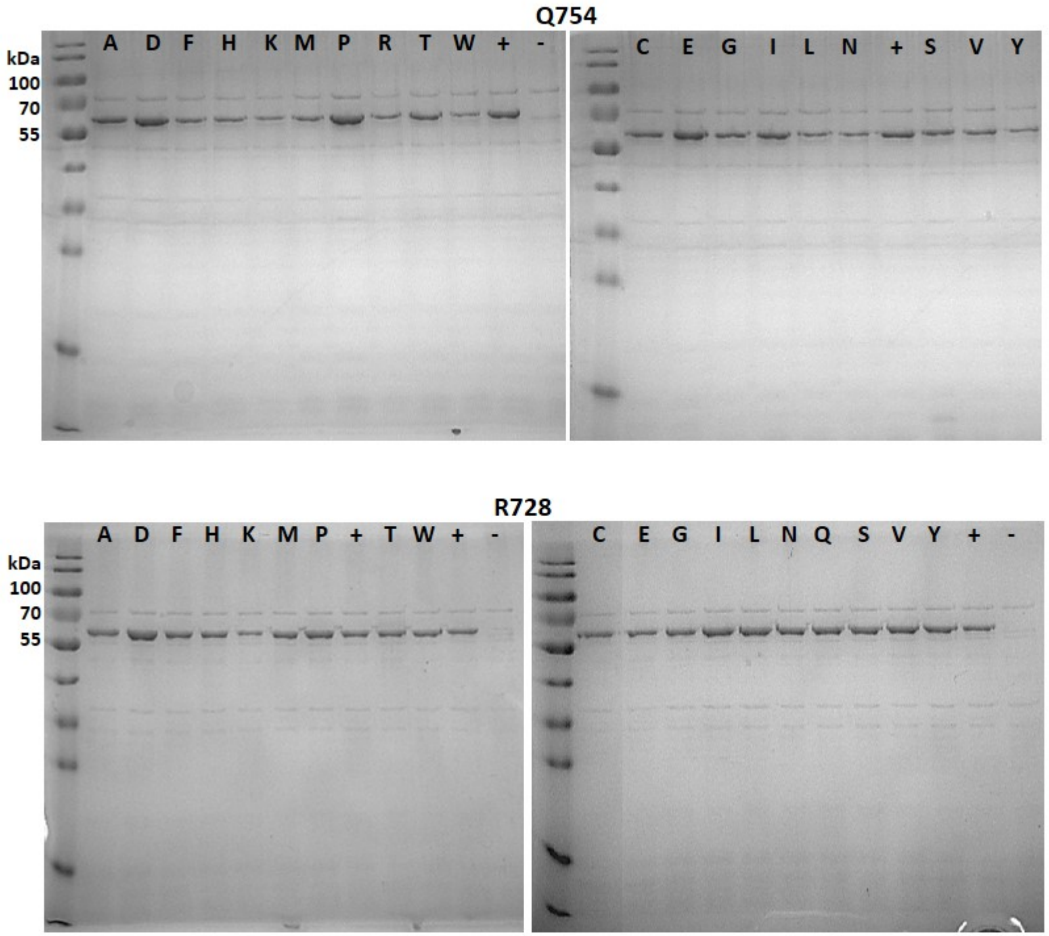

Figure S1.

Expression profile of target sites Q754 (upper panel) and R728 (lower panel). 19 single site mutants for each position were overexpressed, and heat denatured soluble fraction were analysed on SDS-PAGE gel. The mutants are represented in single letter notation of amino acids. “+” signifies wild type KlenTaq DNA polymerase, “-” plasmid control without insert.

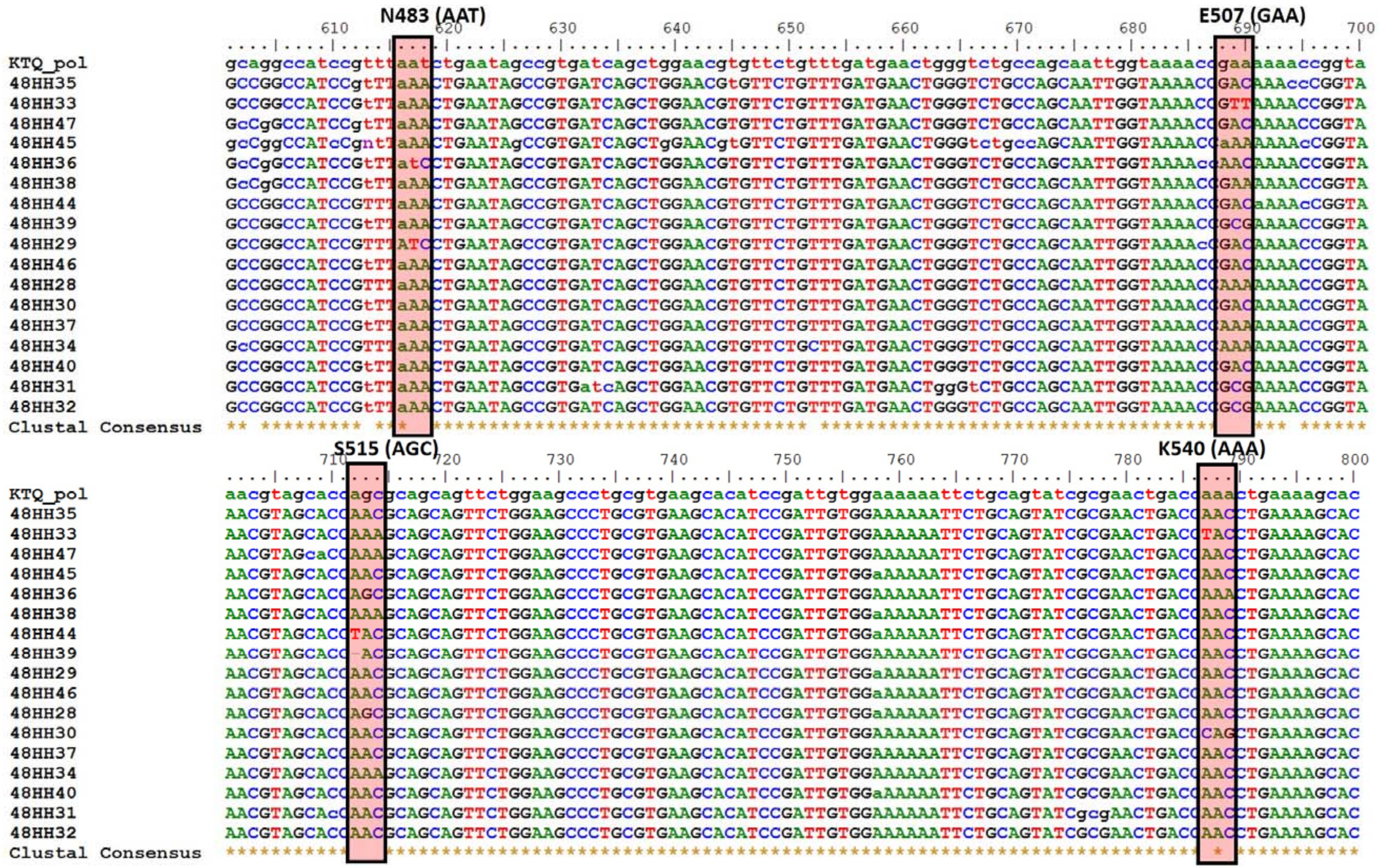

N483 (AAT)
E507 (GAA)
S515 (AGC)
K540 (AAA)
KTQ_pol
48HH35
48HH33
48HH47
48HH45
48HH36
48HH38
48HH44
48HH39
48HH29
48HH46
48HH28
48HH30
48HH37
48HH34
48HH40
48HH31
48HH32
Clustal Consensus

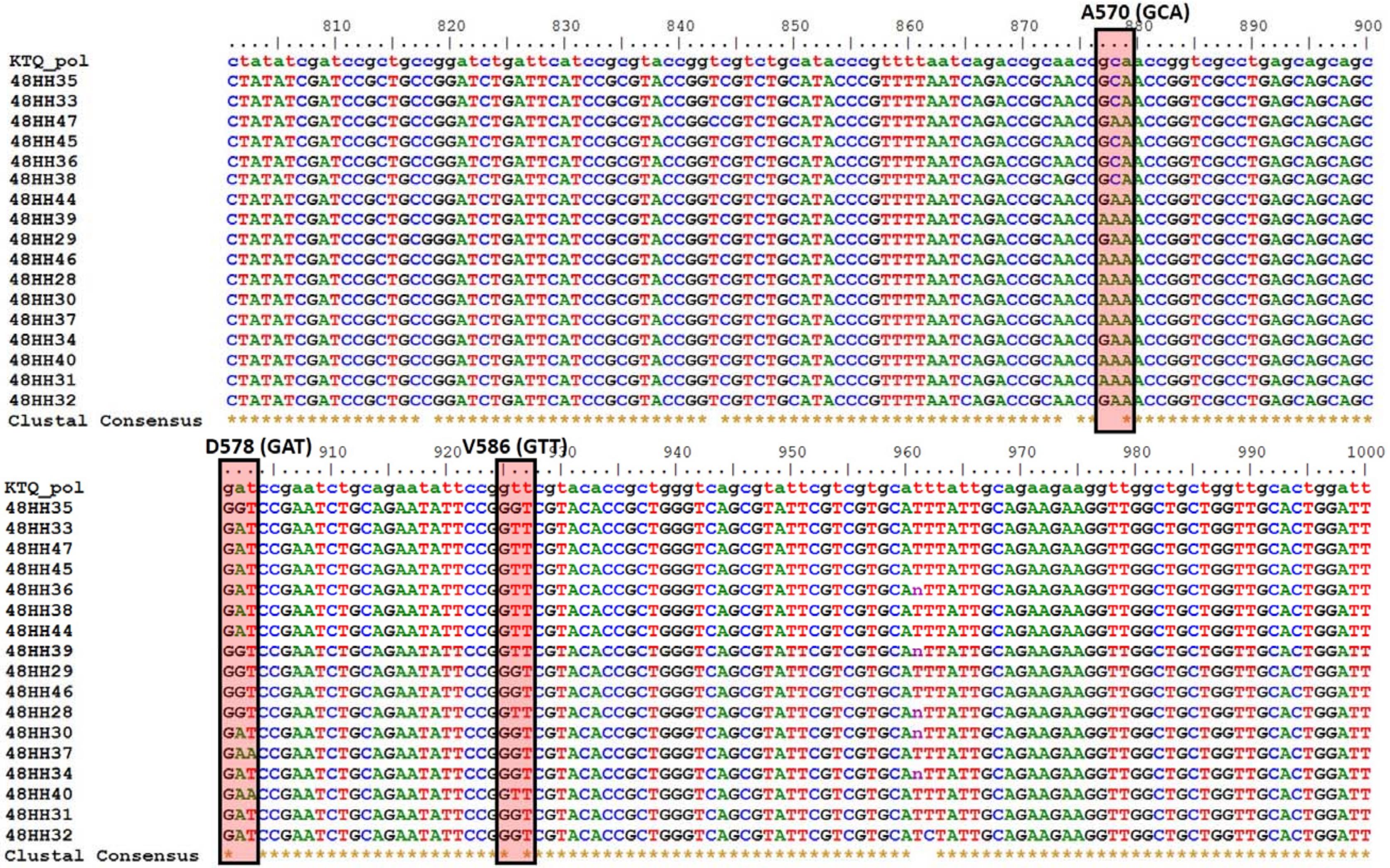
A570 (GCA)
D578 (GAT)
V586 (GTT)
KTQ_pol
48HH35
48HH33
48HH47
48HH45
48HH36
48HH38
48HH44
48HH39
48HH29
48HH46
48HH28
48HH30
48HH37
48HH34
48HH40
48HH31
48HH32
Clustal Consensus

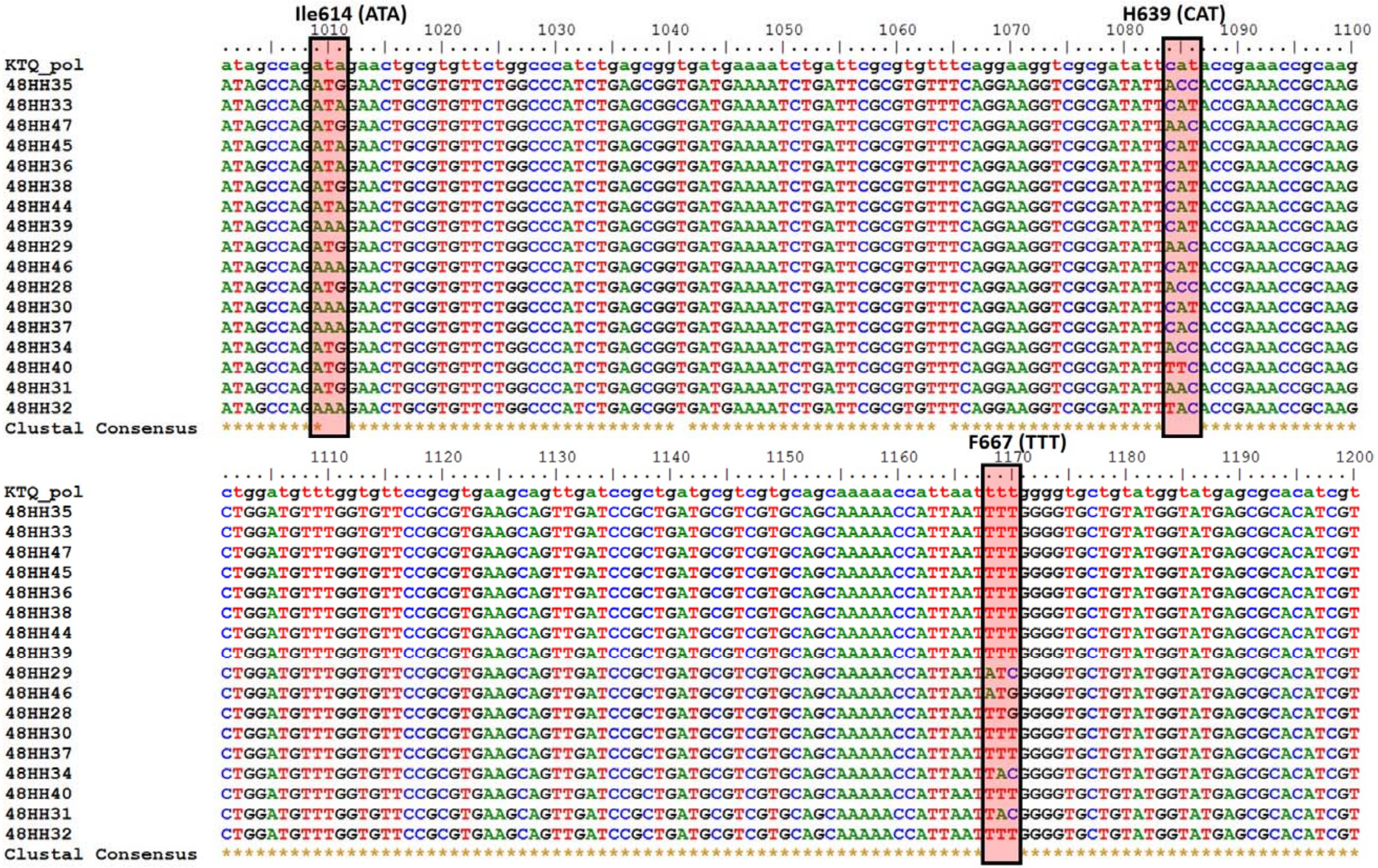
Ile614 (ATA)
H639 (CAT)
1010 1020 1030 1040 1050 1060 1070 1080 1090 1100
KTQ_pol atagccagatagaactgcgtgttctggcccatctgagcggtgatgaaaatctgattcgcgtgtttcaggaaggtcgcgatattcataccgaaaccgcaag
48HH35 ATAGCCAGATGGAACTGCGTGTTCTGGCCCATCTGAGCGGTGATGAAAATCTGATTCGCGTGTTTCAGGAAGGTCGCGATATTACCACCGAAACCGCAAG
48HH33 ATAGCCAGATAGAACTGCGTGTTCTGGCCCATCTGAGCGGCGATGAAAATCTGATTCGCGTGTTTCAGGAAGGTCGCGATATTCATACCGAAACCGCAAG
48HH47 ATAGCCAGATGGAACTGCGTGTTCTGGCCCATCTGAGCGGTGATGAAAATCTGATTCGCGTGTCTCAGGAAGGTCGCGATATTAACACCGAAACCGCAAG
48HH45 ATAGCCAGATAGAACTGCGTGTTCTGGCCCATCTGAGCGGTGATGAAAATCTGATTCGCGTGTTTCAGGAAGGTCGCGATATTCATACCGAAACCGCAAG
48HH36 ATAGCCAGATAGAACTGCGTGTTCTGGCCCATCTGAGCGGTGATGAAAATCTGATTCGCGTGTTTCAGGAAGGTCGCGATATTCATACCGAAACCGCAAG
48HH38 ATAGCCAGATGGAACTGCGTGTTCTGGCCCATCTGAGCGGTGATGAAAATCTGATTCGCGTGTTTCAGGAAGGTCGCGATATTCATACCGAAACCGCAAG
48HH44 ATAGCCAGATAGAACTGCGTGTTCTGGCCCATCTGAGCGGTGATGAAAATCTGATTCGCGTGTTTCAGGAAGGTCGCGATATTCATACCGAAACCGCAAG
48HH39 ATAGCCAGAAAGAACTGCGTGTTCTGGCCCATCTGAGCGGTGATGAAAATCTGATTCGCGTGTTTCAGGAAGGTCGCGATATTCATACCGAAACCGCAAG
48HH29 ATAGCCAGATGGAACTGCGTGTTCTGGCCCATCTGAGCGGTGATGAAAATCTGATTCGCGTGTTTCAGGAAGGTCGCGATATTAACACCGAAACCGCAAG
48HH46 ATAGCCAGAAAGAACTGCGTGTTCTGGCCCATCTGAGCGGTGATGAAAATCTGATTCGCGTGTTTCAGGAAGGTCGCGATATTCATACCGAAACCGCAAG
48HH28 ATAGCCAGATGGAACTGCGTGTTCTGGCCCATCTGAGCGGTGATGAAAATCTGATTCGCGTGTTTCAGGAAGGTCGCGATATTACCACCGAAACCGCAAG
48HH30 ATAGCCAGAAAGAACTGCGTGTTCTGGCCCATCTGAGCGGTGATGAAAATCTGATTCGCGTGTTTCAGGAAGGTCGCGATATTCATACCGAAACCGCAAG
48HH37 ATAGCCAGAAAGAACTGCGTGTTCTGGCCCATCTGAGCGGTGATGAAAATCTGATTCGCGTGTTTCAGGAAGGTCGCGATATTCACACCGAAACCGCAAG
48HH34 ATAGCCAGATGGAACTGCGTGTTCTGGCCCATCTGAGCGGTGATGAAAATCTGATTCGCGTGTTTCAGGAAGGTCGCGATATTACCACCGAAACCGCAAG
48HH40 ATAGCCAGATGGAACTGCGTGTTCTGGCCCATCTGAGCGGTGATGAAAATCTGATTCGCGTGTTTCAGGAAGGTCGCGATATTTTCACCGAAACCGCAAG
48HH31 ATAGCCAGATGGAACTGCGTGTTCTGGCCCATCTGAGCGGTGATGAAAATCTGATTCGCGTGTTTCAGGAAGGTCGCGATATTAACACCGAAACCGCAAG
48HH32 ATAGCCAGAAAGAACTGCGTGTTCTGGCCCATCTGAGCGGTGATGAAAATCTGATTCGCGTGTTTCAGGAAGGTCGCGATATTTACACCGAAACCGCAAG
Clustal Consensus
F667 (TTT)
1110 1120 1130 1140 1150 1160 1170 1180 1190 1200
KTQ_pol ctggatgtttggtgttccgcgtgaagcagttgatccgctgatgcgtcgtgcagcaaaaaccattaattttggggtgctgtatggtatgagcgcacatcgt
48HH35 CTGGATGTTTGGTGTTCCGCGTGAAGCAGTTGATCCGCTGATGCGTCGTGCAGCAAAAACCATTAATTTTGGGGTGCTGTATGGTATGAGCGCACATCGT
48HH33 CTGGATGTTTGGTGTTCCGCGTGAAGCAGTTGATCCGCTGATGCGTCGTGCAGCAAAAACCATTAATTTTGGGGTGCTGTATGGTATGAGCGCACATCGT
48HH47 CTGGATGTTTGGTGTTCCGCGTGAAGCAGTTGATCCGCTGATGCGTCGTGCAGCAAAAACCATTAATTTTGGGGTGCTGTATGGTATGAGCGCACATCGT
48HH45 CTGGATGTTTGGTGTTCCGCGTGAAGCAGTTGATCCGCTGATGCGTCGTGCAGCAAAAACCATTAATTTTGGGGTGCTGTATGGTATGAGCGCACATCGT
48HH36 CTGGATGTTTGGTGTTCCGCGTGAAGCAGTTGATCCGCTGATGCGTCGTGCAGCAAAAACCATTAATTTTGGGGTGCTGTATGGTATGAGCGCACATCGT
48HH38 CTGGATGTTTGGTGTTCCGCGTGAAGCAGTTGATCCGCTGATGCGTCGTGCAGCAAAAACCATTAATTTTGGGGTGCTGTATGGTATGAGCGCACATCGT
48HH44 CTGGATGTTTGGTGTTCCGCGTGAAGCAGTTGATCCGCTGATGCGTCGTGCAGCAAAAACCATTAATTTTGGGGTGCTGTATGGTATGAGCGCACATCGT
48HH39 CTGGATGTTTGGTGTTCCGCGTGAAGCAGTTGATCCGCTGATGCGTCGTGCAGCAAAAACCATTAATTTTGGGGTGCTGTATGGTATGAGCGCACATCGT
48HH29 CTGGATGTTTGGTGTTCCGCGTGAAGCAGTTGATCCGCTGATGCGTCGTGCAGCAAAAACCATTAATATCGGGGTGCTGTATGGTATGAGCGCACATCGT
48HH46 CTGGATGTTTGGTGTTCCGCGTGAAGCAGTTGATCCGCTGATGCGTCGTGCAGCAAAAACCATTAATATGGGGGTGCTGTATGGTATGAGCGCACATCGT
48HH28 CTGGATGTTTGGTGTTCCGCGTGAAGCAGTTGATCCGCTGATGCGTCGTGCAGCAAAAACCATTAATTTGGGGGTGCTGTATGGTATGAGCGCACATCGT
48HH30 CTGGATGTTTGGTGTTCCGCGTGAAGCAGTTGATCCGCTGATGCGTCGTGCAGCAAAAACCATTAATTTTGGGGTGCTGTATGGTATGAGCGCACATCGT
48HH37 CTGGATGTTTGGTGTTCCGCGTGAAGCAGTTGATCCGCTGATGCGTCGTGCAGCAAAAACCATTAATTTTGGGGTGCTGTATGGTATGAGCGCACATCGT
48HH34 CTGGATGTTTGGTGTTCCGCGTGAAGCAGTTGATCCGCTGATGCGTCGTGCAGCAAAAACCATTAATTACGGGGTGCTGTATGGTATGAGCGCACATCGT
48HH40 CTGGATGTTTGGTGTTCCGCGTGAAGCAGTTGATCCGCTGATGCGTCGTGCAGCAAAAACCATTAATTTTGGGGTGCTGTATGGTATGAGCGCACATCGT
48HH31 CTGGATGTTTGGTGTTCCGCGTGAAGCAGTTGATCCGCTGATGCGTCGTGCAGCAAAAACCATTAATTACGGGGTGCTGTATGGTATGAGCGCACATCGT
48HH32 CTGGATGTTTGGTGTTCCGCGTGAAGCAGTTGATCCGCTGATGCGTCGTGCAGCAAAAACCATTAATTTTGGGGTGCTGTATGGTATGAGCGCACATCGT
Clustal Consensus

```
                         1210      1220      1230      1240      1250      1260      1270      1280      1290      1300
                    ....|....|....|....|....|....|....|....|....|....|....|....|....|....|....|....|....|....|....|....|
KTQ_pol             ctgagccaggaactggcaattccgtacgaagaagcccaggcatttatcgaacgttattttcagagctttccgaaagttcgtgcctggattgaaaaaaccc
48HH35              CTGAGCCAGGAACTGGCAATTCCGTACGAAGAAGCCCAGGCATTTATCGAACGTTATTTTCAGAGCTTTCCGAAAGTTCGTGCCTGGATTGAAAAAACCC
48HH33              CTGAGCCAGGAACTGGCAATTCCGTACGAAGAAGCCCAGGCATTTATCGAACGTTATTTTCAGAGCTTTCCGAAAGTTCGTGCCTGGATTGAAAGAACCC
48HH47              CTGAGCCAGGAACTGGCAATTCCGTACGAAGAAGCCCAGGCATTTATCGAACGTTATTTTCAGAGCTTTCCGAAAGTTCGTGCCTGGATTGAAAAAACCC
48HH45              CTGAGCCAGGAACTGGCAATTCCGTACGAAGAAGCCCAGGCATTTATCGAACGTTATTTTCAGAGCTTTCCGAAAGTTCGTGCCTGGATTGAAAAAACCC
48HH36              CTGAGCCAGGAACTGGCAATTCCGTACGAAGAAGCCCAGGCATTTATCGAACGTTATTTTCAGAGCTTTCCGAAAGTTCGTGCCTGGATTGAAAAAACCC
48HH38              CTGAGCCAGGAACTGGCAATTCCGTACGAAGAAGCCCAGGCATTTATCGAACGTTATTTTCAGAGCTTTCCGAAAGTTCGTGCCTGGATTGAAAAAACCC
48HH44              CTGAGCCAGGAACTGGCAATTCCGTACGGAGAAGCCCAGGCATTTATCGAACGTTATTTTCAGAGCTTTCCGAAAGTTCGTGCCTGGATTGAAAAAACCC
48HH39              CTGAGCCAGGAACTGGCAATTCCGTACGAAGAAGCCCAGGCATTTATCGAACGTTATTTTCAGAGCTTTCCGAAAGTTCGTGCCTGGATTGAAAAAACCC
48HH29              CTGAGCCAGGAACTGGCAATTCCGTACGAAGAAGCCCAGGCATTTATCGAACGTTATTTTCAGAGCTTTCCGAAAGTTCGTGCCTGGATTGAAAAAACCC
48HH46              CTGAGCCAGGAACTGGCAATTCCGTACGAAGAAGCCCAGGCATTTATCGAACGTTATTTTCAGAGCTTTCCGAAAGTTCGTGCCTGGATTGAAAAAACCC
48HH28              CTGAGCCAGGAACTGGCAATTCCGTACGAAGAAGCCCAGGCATTTATCGAACGTTATTTTCAGAGCTTTCCGAAAGTTCGTGCCTGGATTGAAAAAACCC
48HH30              CTGAGCCAGGAACTGGCAATTCCGTACGAAGAAGCCCAGGCATTTATCGAACGTTATTTTCAGAGCTTTCCGAAAGTTCGTGCCTGGATTGAAAAAACCC
48HH37              CTGAGCCAGGAACTGGCAATTCCGTACGAAGAAGCCCAGGCATTTATCGAACGTTATTTTCAGAGCTTTCCGAAAGTTCGTGCCTGGATTGAAAAAACCC
48HH34              CTGAGCCAGGAACTGGCAATTCCGTACGAAGAAGCCCAGGCATTTATCGAACGTTATTTTCAGAGCTTTCCGAAAGTTCGTGCCTGGATTGAAAAAACCC
48HH40              CTGAGCCAGGAACTGGCAATTCCGTACGAAGAAGCCCAGGCATTTATCGAACGTTATTTTCAGAGCTTTCCGAAAGTTCGTGCCTGGATTGAAAAAACCC
48HH31              CTGAGCCAGGAACTGGCAATTCCGTACGAAGAAGCCCAGGCATTTATCGAACGTTATTTTCAGAGCTTTCCGAAAGTTCGTGCCTGGATTGAAAAAACCC
48HH32              CTGAGCCAGGAACTGGCAATTCCGTACGAAGAAGCCCAGGCATTTATCGAACGTTATTTTCAGAGCTTTCCGAAAGTTCGTGCCTGGATTGAAAAAACCC
Clustal Consensus   **************************** ***************************************************************** *****

                         1310      1320      1330      1340      1350      1360      1370      1380      1390      1400
                    ....|....|....|....|....|....|....|....|....|....|....|....|....|....|....|....|....|....|....|
KTQ_pol             tggaagaaggtcgtcgtcgcggttatgttgaaaccctgtttggtcgtcgtcgttatgttccggatctggaagcacgtgttaaaagcgttcgtgaagcagc
48HH35              TGGAAGAAGGTCGTCGTCGCGGTTATGTTGAAACCCTGTTTGGTCGTCGTCGTTATGTTCCGGATCTGGAAGCACGTGTTAAAAGCGTTCGTGAAGCAGC
48HH33              TGGAAGAAGGTCGTCGTCGCGGTTATGTTGAAACCCTGTTTGGTCGTCGTCGTTATGTTCCGGATCTGGAAGCACGTGTTAAAAGCGTTCGTGAAGCAGC
48HH47              TGGAAGAAGGTCGTCGTCGCGGTTATGTTGAAACCCTGTTTGGTCGTCGTCGTTATGTTCCGGATCTGGAAGCACGTGTTAAAAGCGTTCGTGAAGCAGC
48HH45              TGGAAGAAGGTCGTCGTCGCGGTTATGTTGAAACCCTGTTTGGTCGTCGTCGTTATGTTCCGGATCTGGAAGCACGTGTTAAAAGCGTTCGTGAAGCAGC
48HH36              TGGAAGAAGGTCGTCGTCGCGGTTATGTTGAAACCCTGTTTGGTCGTCGTCGTTATGTTCCGGATCTGGAAGCACGTGTTAAAAGCGTTCGTGAAGCAGC
48HH38              TGGAAGAAGGTCGTCGTCGCGGTTATGTTGAAACCCTGTTTGGTCGTCGTCGTTATGTTCCGGATCTGGAAGCACGTGTTAAAAGCGTTCGTGAAGCAGC
48HH44              TGGAAGAAGGTCGTCGTCGCGGTTATGTTGAAACCCTGTTTGGTCGTCGTCGTTATGTTCCGGATCTGGAAGCACGTGTTAAAAGCGTTCGTGAAGCAGC
48HH39              TGGAAGAAGGTCGTCGTCGCGGTTATGTTGAAACCCTGTTTGGTCGTCGTCGTTATGTTCCGGATCTGGAAGCACGTGTTAAAAGCGTTCGTGAAGCAGC
48HH29              TGGAAGAAGGTCGTCGTCGCGGTTATGTTGAAACCCTGTTTGGTCGTCGTCGTTATGTTCCGGATCTGGAAGCACGTGTTAAAAGCGTTCGTGAAGCAGC
48HH46              TGGAAGAAGGTCGTCGTCGCGGTTATGTTGAAACCCTGTTTGGTCGTCGTCGTTATGTTCCGGATCTGGAAGCACGTGTTAAAAGCGCTCGTGAAGCAGC
48HH28              TGGAAGAAGGTCGTCGTCGCGGTTATGTTGAAACCCTGTTTGGTCGTCGTCGTTATGTTCCGGATCTGGAAGCACGTGTTAAAAGCGTTCGTGAAGCAGC
48HH30              TGGAAGAAGGTCGTCGTCGCGGTTATGTTGAAACCCTGTTTGGTCGTCGTCGTTATGTTCCGGATCTGGAAGCACGTGTTAAAAGCGTTCGTGAAGCAGC
48HH37              TGGAAGAAGGTCGTCGTCGCGGTTATGTTGAAACCCTGTTTGGTCGTCGTCGTTATGTTCCGGATCTGGAAGCACGTGTTAAAAGCGTTCGTGAAGCAGC
48HH34              TGGAAGAAGGTCGTCGTCGCGGTTATGTTGAAACCCTGTTTGGTCGTCGTCGTTATGTTCCGGATCTGGAAGCACGTGTTAAAAGCGTTCGTGAAGCAGC
48HH40              TGGAAGAAGGTCGTCGTCGCGGTTATGTTGAAACCCTGTTTGGTCGTCGTCGTTATGTTCCGGATCTGGAAGCACGTGTTAAAAGCGTTCGTGAAGCAGC
48HH31              TGGAAGAAGGTCGTCGTCGCGGTTATGTTGAAACCCTGTTTGGTCGTCGTCGTTATGTTCCGGATCTGGAAGCACGTGTTAAAAGCGTTCGTGAAGCAGC
48HH32              TGGAAGAAGGTCGTCGTCGCGGTTATGTTGAAACCCTGTTTGGTCGTCGTCGTTATGTTCCGGATCTGGAAGCACGTGTTAAAAGCGTTCGTGAAGCAGC
Clustal Consensus   *************************************************************************************** ************
```

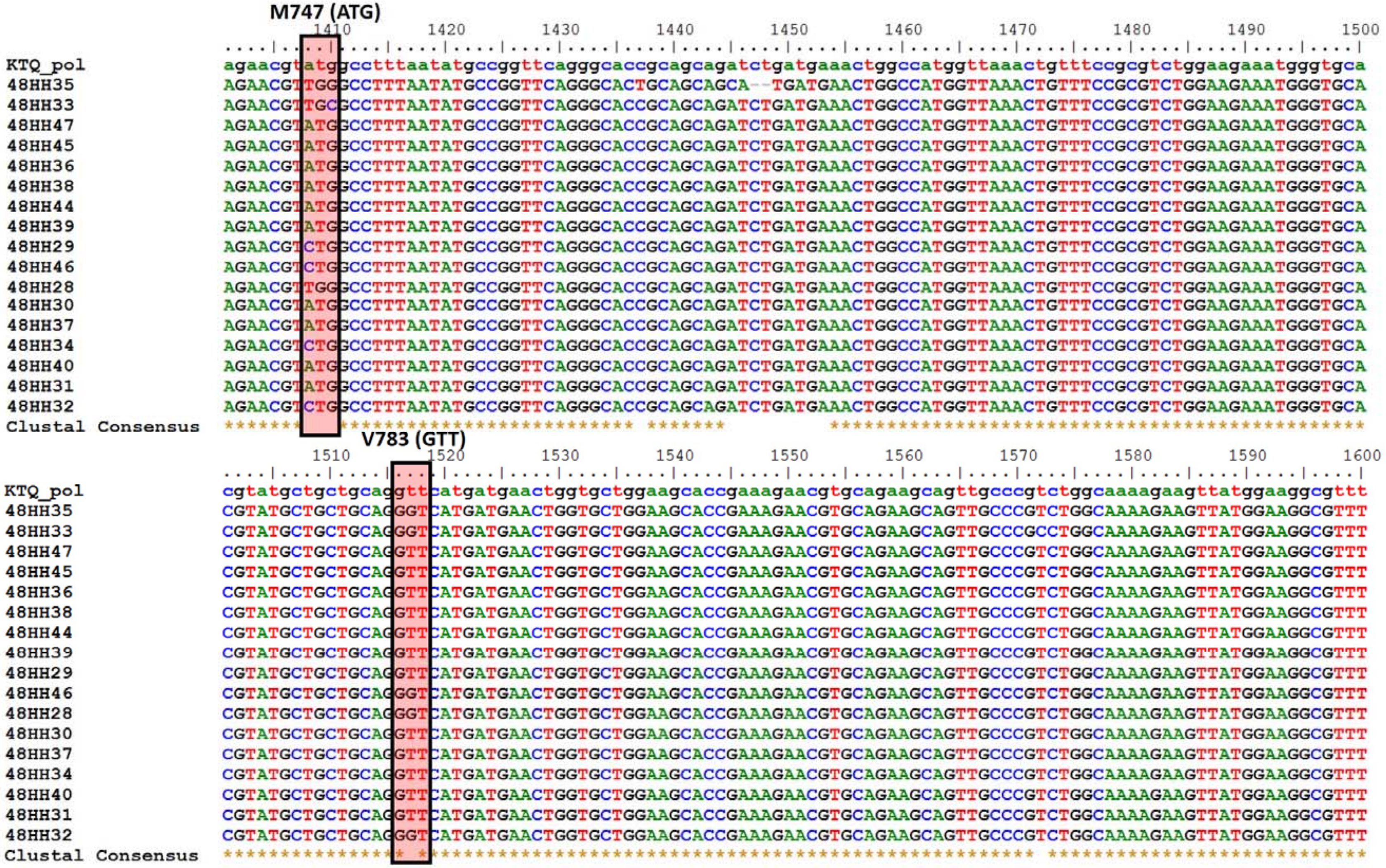

Figure S2.

Multiple sequence alignment of random 20 clones from the combinatorial library constructed through shuffling of 173 PCR active mutants. Wild type KlenTaq DNA polymerase nucleotide sequence was also included in alignment. The mutational positions are labelled in single letter notation of amino acids with their respective wild type codons in brackets. Black solid line boxes with red shade highlights the change of codons in respective clones. Note all the positions got shuffled even for positions H639 and F667 with less number of active mutants. Ruler in the alignment is arbitrary number.

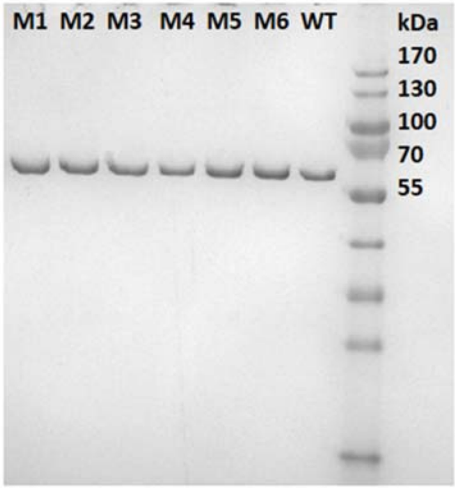

Figure S3.

SDS-PAGE gel of affinity purified wild type KlenTaq DNA polymerase and selected positive hits from allelic discrimination screening. Mutants were labelled as M1, M2, M3, M4, M5, M6 and wild type KlenTaq DNA polymerase as WT.

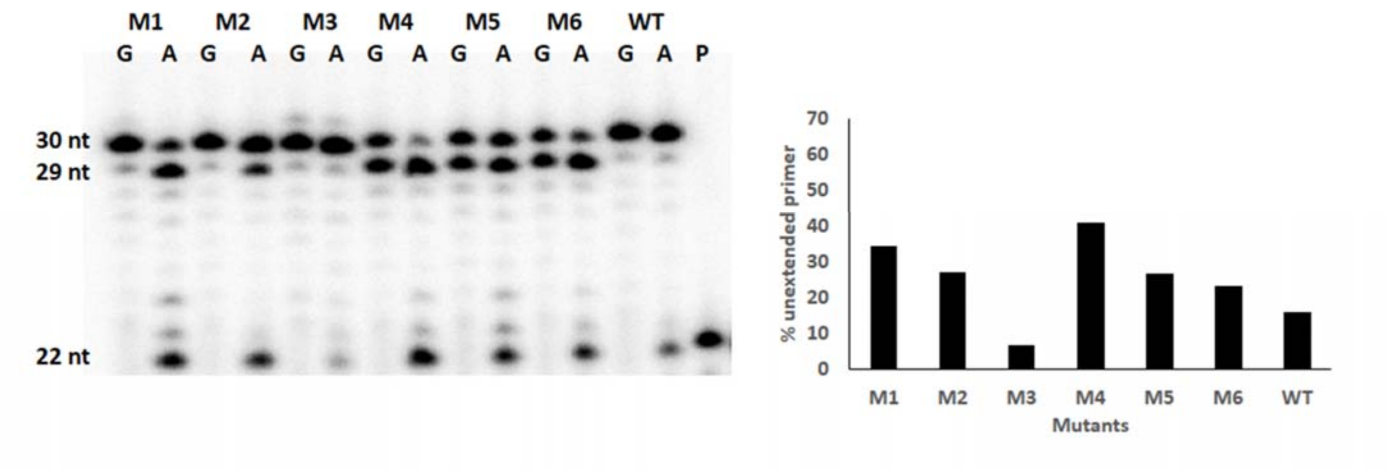

Figure S4.

Primer extension assay of selected mutants purified to homogenity along with wild type KlenTaq DNA polymerase. Mutants were labelled as M1, M2, M3, M4, M5, M6 and wild type KlenTaq DNA polymerase as WT. 30nM purified enzymes were applied in matched (G) and mismatched (A) reaction containing 100µM dNTPs in 1X KlenTaq reaction buffer and the primer extension was stopped after 10min (Left). Plot showing the percentage of mismatched (A) primer remain unextended in mismatch primer template reaction. Among mutants tested, mutant M4 had 40% of mismatch primer remained unextended whereas WT had only 16% primer unextended. M4 was characterized further and named as Mut_ADL (Right).

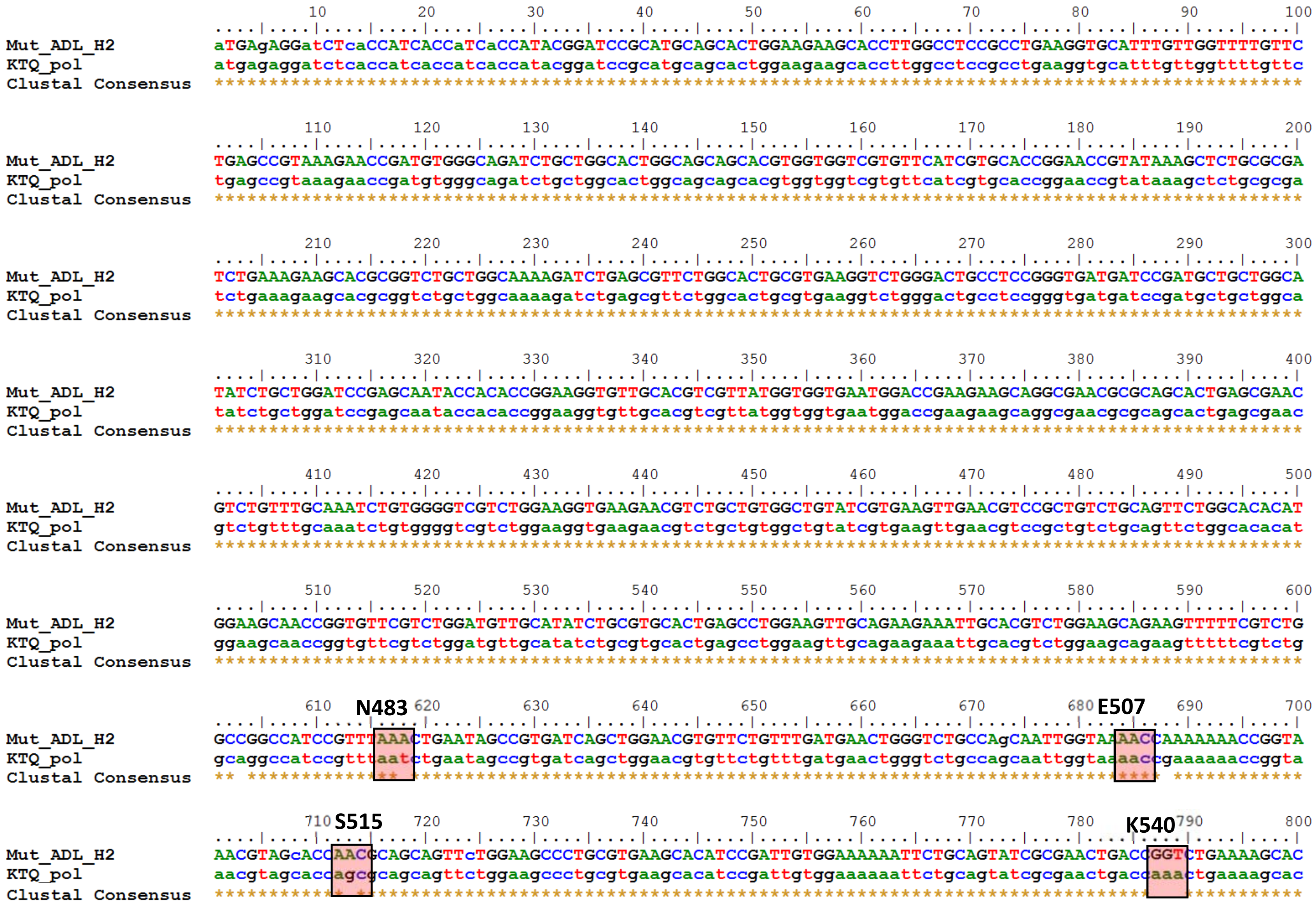
10 20 30 40 50 60 70 80 90 100
Mut_ADL_H2 aTGAgAGGatCTcaCCATCACCaTCaCCATACGGATCCGCATGCAGCACTGGAAGAAGCACCTTGGCCTCCGCCTGAAGGTGCATTTGTTGGTTTTGTTC
KTQ_pol atgagaggatctcaccatcaccatcaccatacggatccgcatgcagcactggaagaagcaccttggcctccgcctgaaggtgcatttgttggttttgttc
Clustal Consensus **
110 120 130 140 150 160 170 180 190 200
Mut_ADL_H2 TGAGCCGTAAAGAACCGATGTGGGCAGATCTGCTGGCACTGGCAGCAGCACGTGGTGGTCGTGTTCATCGTGCACCGGAACCGTATAAAGCTCTGCGCGA
KTQ_pol tgagccgtaaagaaccgatgtgggcagatctgctggcactggcagcagcacgtggtggtcgtgttcatcgtgcaccggaaccgtataaagctctgcgcga
Clustal Consensus **
210 220 230 240 250 260 270 280 290 300
Mut_ADL_H2 TCTGAAAGAAGCACGCGGTCTGCTGGCAAAAGATCTGAGCGTTCTGGCACTGCGTGAAGGTCTGGGACTGCCTCCGGGTGATGATCCGATGCTGCTGGCA
KTQ_pol tctgaaagaagcacgcggtctgctggcaaaagatctgagcgttctggcactgcgtgaaggtctgggactgcctccgggtgatgatccgatgctgctggca
Clustal Consensus **
310 320 330 340 350 360 370 380 390 400
Mut_ADL_H2 TATCTGCTGGATCCGAGCAATACCACACCGGAAGGTGTTGCACGTCGTTATGGTGGTGAATGGACCGAAGAAGCAGGCGAACGCGCAGCACTGAGCGAAC
KTQ_pol tatctgctggatccgagcaataccacaccggaaggtgttgcacgtcgttatggtggtgaatggaccgaagaagcaggcgaacgcgcagcactgagcgaac
Clustal Consensus **
410 420 430 440 450 460 470 480 490 500
Mut_ADL_H2 GTCTGTTTGCAAATCTGTGGGGTCGTCTGGAAGGTGAAGAACGTCTGCTGTGGCTGTATCGTGAAGTTGAACGTCCGCTGTCTGCAGTTCTGGCACACAT
KTQ_pol gtctgtttgcaaatctgtggggtcgtctggaaggtgaagaacgtctgctgtggctgtatcgtgaagttgaacgtccgctgtctgcagttctggcacacat
Clustal Consensus **
510 520 530 540 550 560 570 580 590 600
Mut_ADL_H2 GGAAGCAACCGGTGTTCGTCTGGATGTTGCATATCTGCGTGCACTGAGCCTGGAAGTTGCAGAAGAAATTGCACGTCTGGAAGCAGAAGTTTTTCGTCTG
KTQ_pol ggaagcaaccggtgttcgtctggatgttgcatatctgcgtgcactgagcctggaagttgcagaagaaattgcacgtctggaagcagaagtttttcgtctg
Clustal Consensus **
610 N483 620 630 640 650 660 670 680 E507 690 700
Mut_ADL_H2 GCCGGCCATCCGTTTAAACTGAATAGCCGTGATCAGCTGGAACGTGTTCTGTTTGATGAACTGGGTCTGCCAgCAATTGGTAAAACCAAAAAAACCGGTA
KTQ_pol gcaggccatccgtttaatctgaatagccgtgatcagctggaacgtgttctgtttgatgaactgggtctgccagcaattggtaaaaccgaaaaaaccggta
Clustal Consensus ** ************** *** ************
710 S515 720 730 740 750 760 770 780 K540 790 800
Mut_ADL_H2 AACGTAGcACCAACGCAGCAGTTcTGGAAGCCCTGCGTGAAGCACATCCGATTGTGGAAAAAATTCTGCAGTATCGCGAACTGACCGGTCTGAAAAGCAC
KTQ_pol aacgtagcaccagcgcagcagttctggaagccctgcgtgaagcacatccgattgtggaaaaaattctgcagtatcgcgaactgaccaaactgaaaagcac
Clustal Consensus ************ **   ************

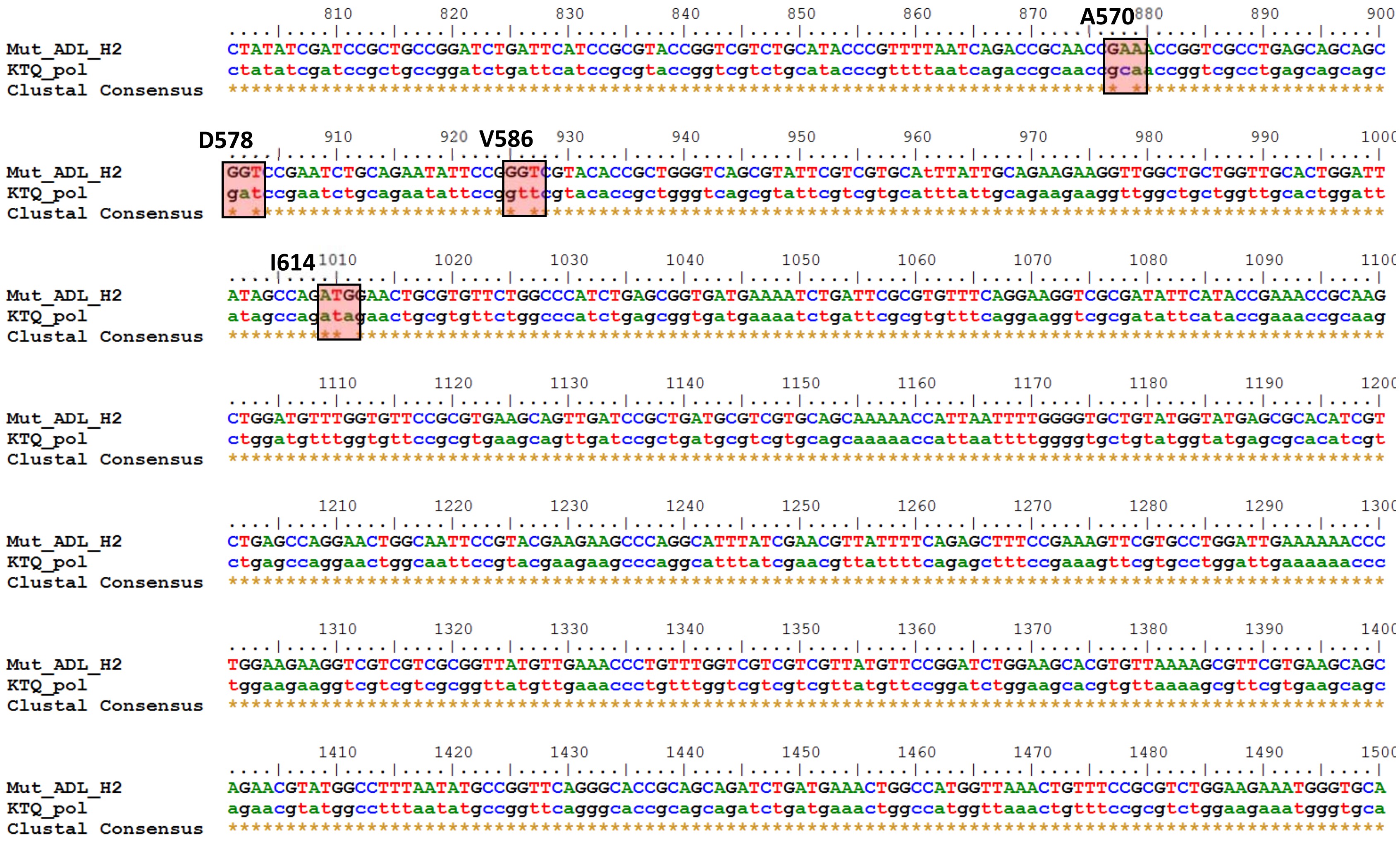
A570
D578
V586
I614
Mut_ADL_H2
KTQ_pol
Clustal Consensus

```
                           1510      1520      1530      1540      1550      1560      1570      1580      1590      1600
                   ....|....|....|....|....|....|....|....|....|....|....|....|....|....|....|....|....|....|....|....|
Mut_ADL_H2         CGTATGCTGCTGCAGGTTCATGATGAACTGGTGCTGGAAGCACCGAAAGAACGTGCAGAAGCAGTTGCCCGTCTGGCAAAAGAAGTTATGGAAGGCGTTT
KTQ_pol            cgtatgctgctgcaggttcatgatgaactggtgctggaagcaccgaaagaacgtgcagaagcagttgcccgtctggcaaaagaagttatggaaggcgttt
Clustal Consensus  ****************************************************************************************************

                           1610      1620      1630      1640      1650      1660      1670
                   ....|....|....|....|....|....|....|....|....|....|....|....|....|....|
Mut_ADL_H2         AtCCGCTGGCAGTTCCGCTGGAAGTTGAAGTTGGTATTGGTGAAGATTGGCTGTCTGCaAAAGaAtaaAA
KTQ_pol            atccgctggcagttccgctggaagttgaagttggtattggtgaagattggctgtctgcaaaagaa-----
Clustal Consensus  *****************************************************************
```

Figure S5.

Multiple sequence alignment of wild type KlenTaq DNA polymerase and Mut_ADL. The difference in nucleotide sequence at the target sites are marked in red box with solid black line. The position of target residues that got mutated were marked above the red boxes. The numbering in alignment ruler are arbitary.

A

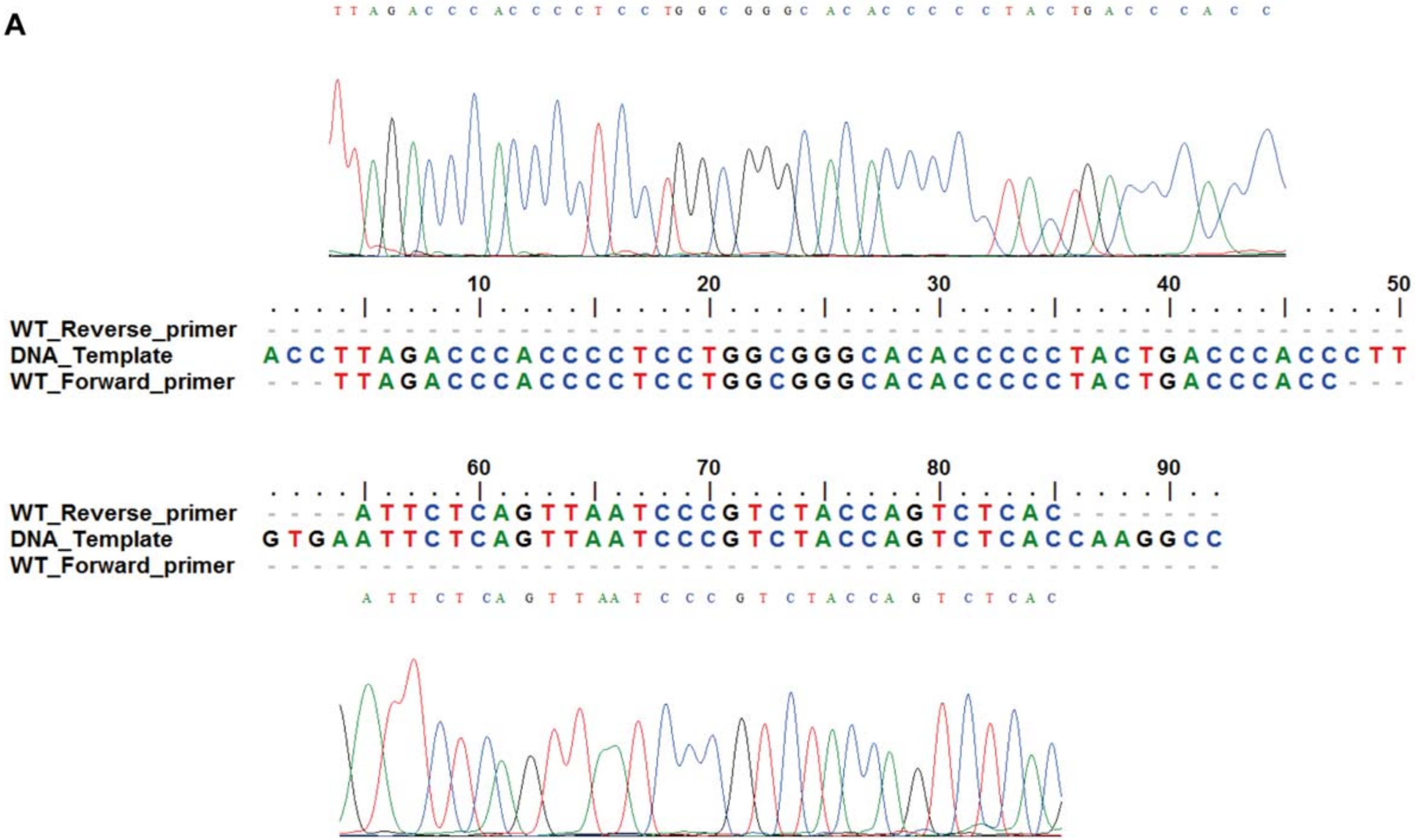
10 20 30 40 50
WT_Reverse_primer
DNA_Template ACCTTAGACCCACCCCTCCTGGCGGGCACACCCCCTACTGACCCACCCTT
WT_Forward_primer TTAGACCCACCCCTCCTGGCGGGCACACCCCCTACTGACCCACC
60 70 80 90
WT_Reverse_primer ATTCTCAGTTAATCCCGTCTACCAGTCTCAC
DNA_Template GTGAATTCTCAGTTAATCCCGTCTACCAGTCTCACCAAGGCC
WT_Forward_primer

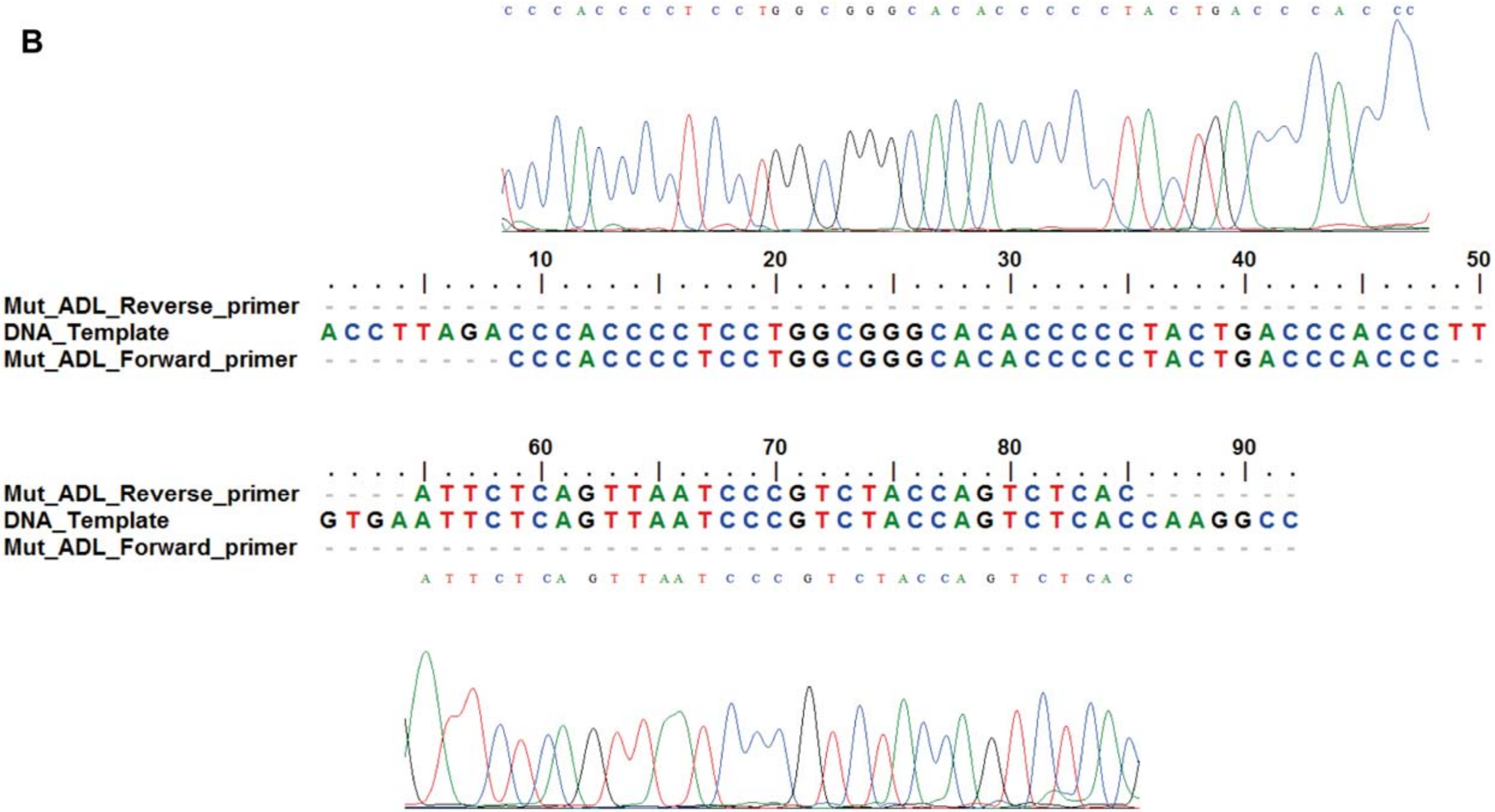

Figure S6. Sanger sequencing analysis of amplified products. The products that were amplified by respective enzymes (WT, Mut_ADL and Mut_RT) were presented in sequence alignment. Section of electropherogram of sequencing reactions with forward primer [5'-d(CTT GGT GAG ACT GGT AGA CG)-3'] (above sequence alignment) and reverse primer [5'-d(TTA GAC CCA CCC CTC CTG GCG)-3'] (below sequence alignment) respectively were shown. A) WT – Amplificate from DNA dependent DNA synthesis B) Mut_ADL - Amplificate from DNA dependent DNA synthesis.

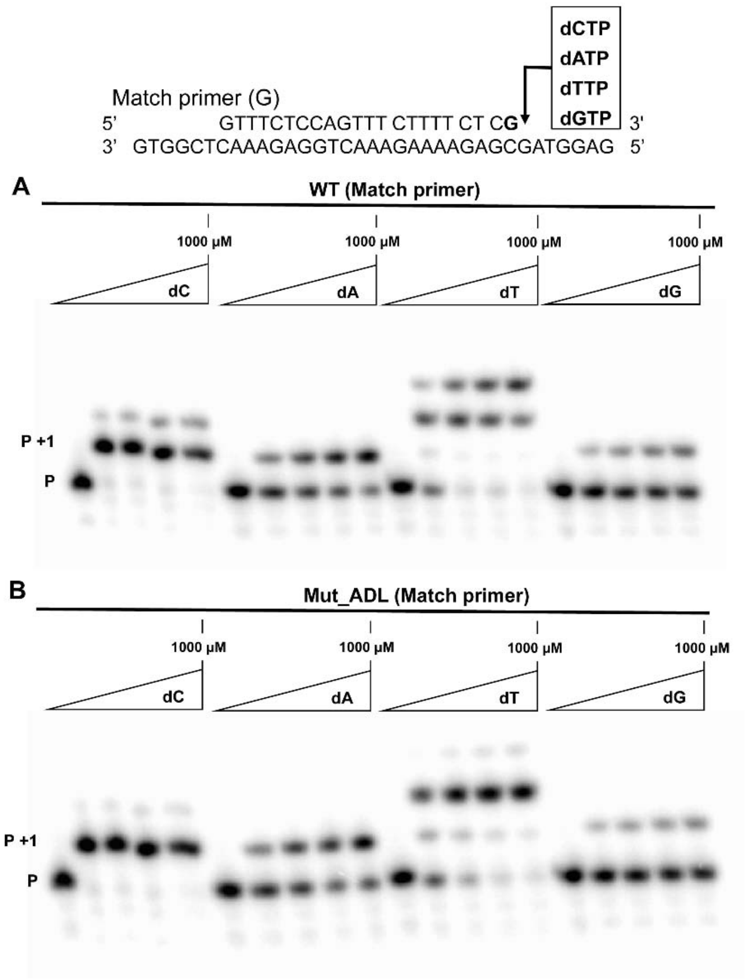

Figure S7. Single nucleotide misinsertion assay for wild type KlenTaq DNA polymerase and Mut_ADL. The reaction (20 µl) contained 1X KlenTaq reaction buffer, 250 nM template (5'-d(GAG GTA GCG AGA AAA GAA ACT GGA GAA ACT CGG TG)3'), 150 nM of 5' radioactively labelled Match primer (5'-d(GTT TCT CCA GTT TCT TTT CTC G)3'). After pre-incubation at 95°C for 2 min, the reaction mixture was cooled to 55°C. The reaction was performed in presence of 10 nM of enzyme and individual all four nucleotides (0, 200, 400, 600, 1000 µM). The reactions were allowed to proceed for 10 min and terminated with 2 volumes of stopping solution (80% [v/v] formamide, 20 mM EDTA, 0.25% [w/v] bromophenol blue, 0.25% [w/v] xylene cyanol). 'P' indicates the unextended primer and 'P + 1' denotes the primer extended by one nucleotide, and so forth. Full-length gel is given in Supplementary Fig. S18.

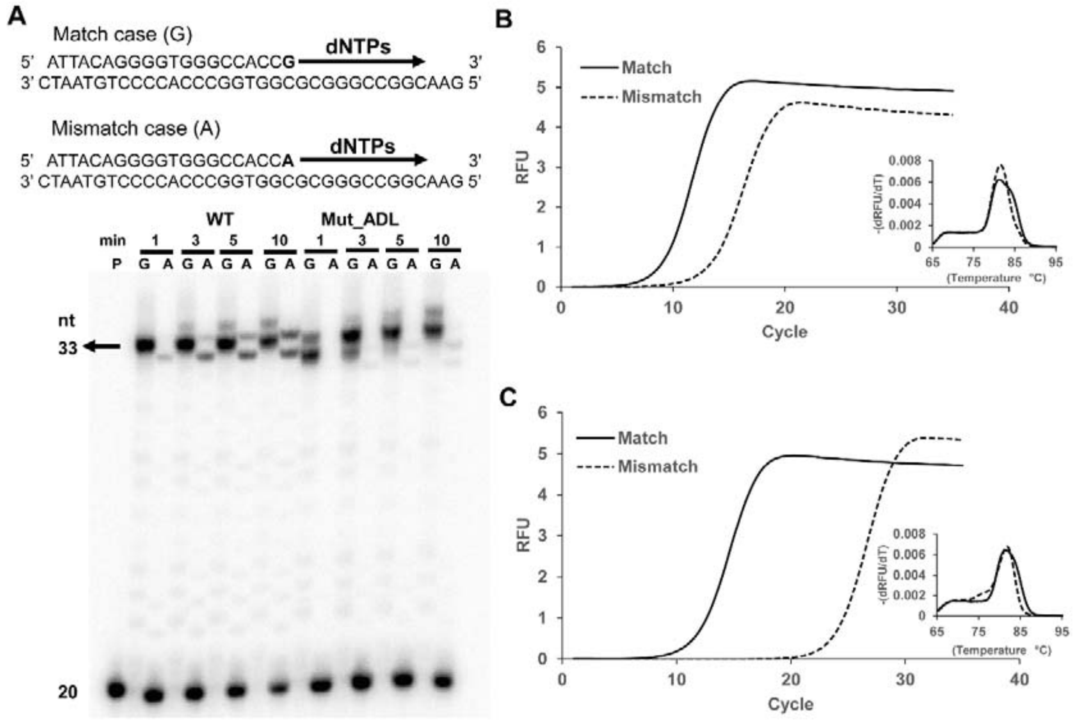

Figure S8. Improved allelic discrimination on different sequence context. (**A**) Sequence of employed primer-template pair. The 3' end of primer either terminates with a matched (G) or mismatched (A) nucleotide. Primer extension reaction was performed as indicated in material section for allelic discrimination, by employing the indicated DNA substrates along with wild-type (WT) and mutant DNA polymerase Mut_ADL, respectively. Reactions were stopped at different time points, as indicated. Full-length gel is given in Supplementary Fig. S19. (**B, C**) Real time PCR reaction (20 µl) contained 1X KlenTaq reaction buffer, 200 µM dNTPs,50 mM betaine (Sigma), 50 nM Taq DNA polymerase aptamer [5'-d(CGA TCA TCT CAG AAC ATT CTT AGC GTT TTG TTC TTG TGT ATG ATC G)3'] and 0.6X SYBR® Green I (Sigma). As template, 139nt [sequence of promoter region of Homo sapiens Nanog homeodomain transcription factor (NANOG) gene][1] 100 pM DNA template_1 (sequence in Supplementary Table S1) was used with 200 nM primer either Match_case [5'-d(ATT ACA GGG GTG GGC CAC CG) 3'] or Mismatch_case [5'-d(ATT ACA GGG GTG GGC CAC CA) 3'] and 200 nM Rev_primer [5'-d(GGT TCA CCA CGT TTC CAA CTT TTA AAT CAG G) 3'] in two separate reactions. After an initial denaturation at 95°C for 1 min, two step thermal cycling was repeated for 35 cycles (95°C for 10 s and 68°C for 60 s) and the reaction was analysed by real-time PCR curves and melting curve measurement within the Light Cycler® 96 instrument (Roche). Real time PCR experiments with purified enzymes (200 nM), showing the discrimination property of (**B**) wild-type KlenTaq DNA polymerase and (**C**) mutant DNA polymerase Mut_ADL respectively. Insert plot showing their corresponding melting peaks.

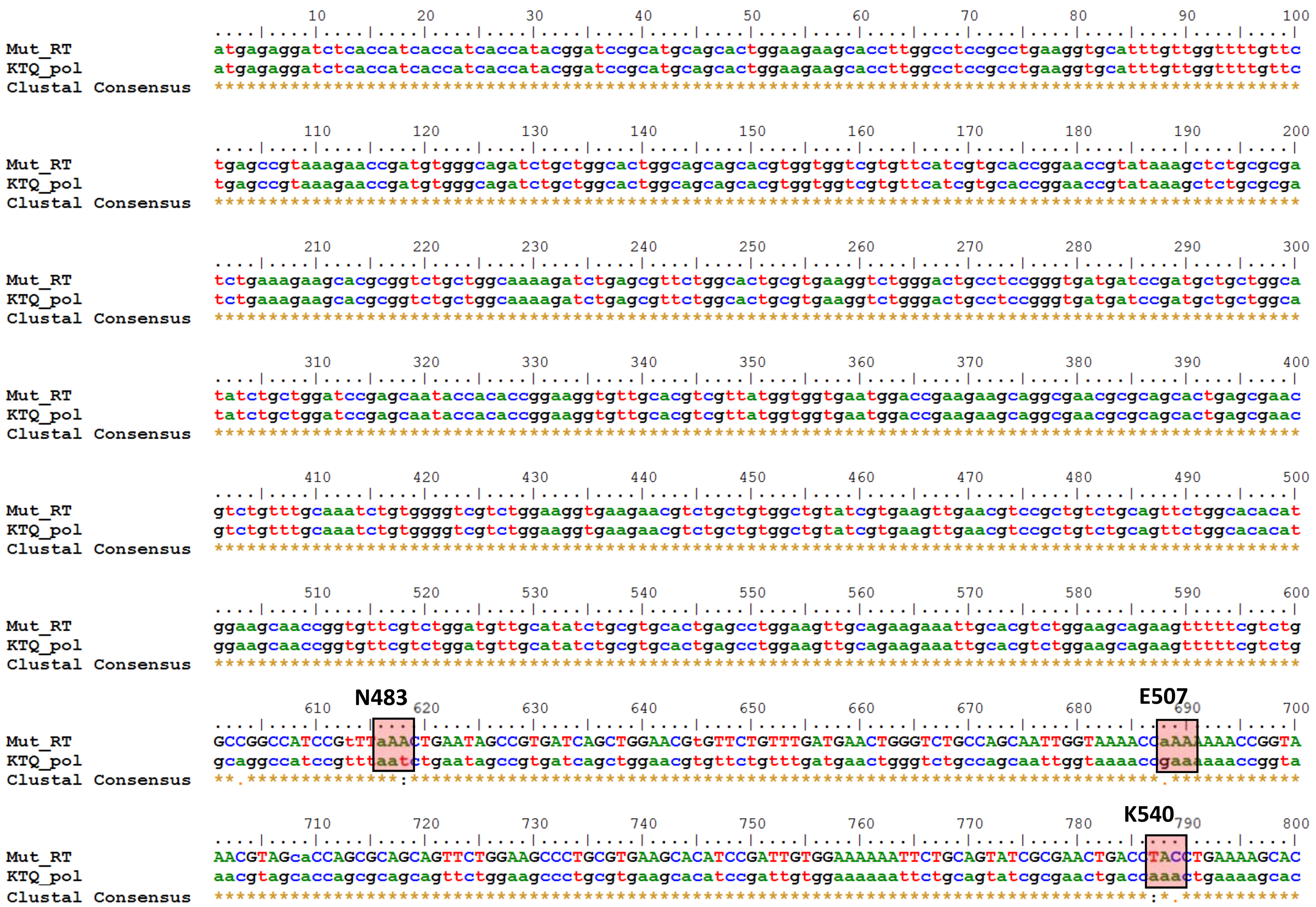

Mut_RT
KTQ_pol
Clustal Consensus
N483
E507
K540

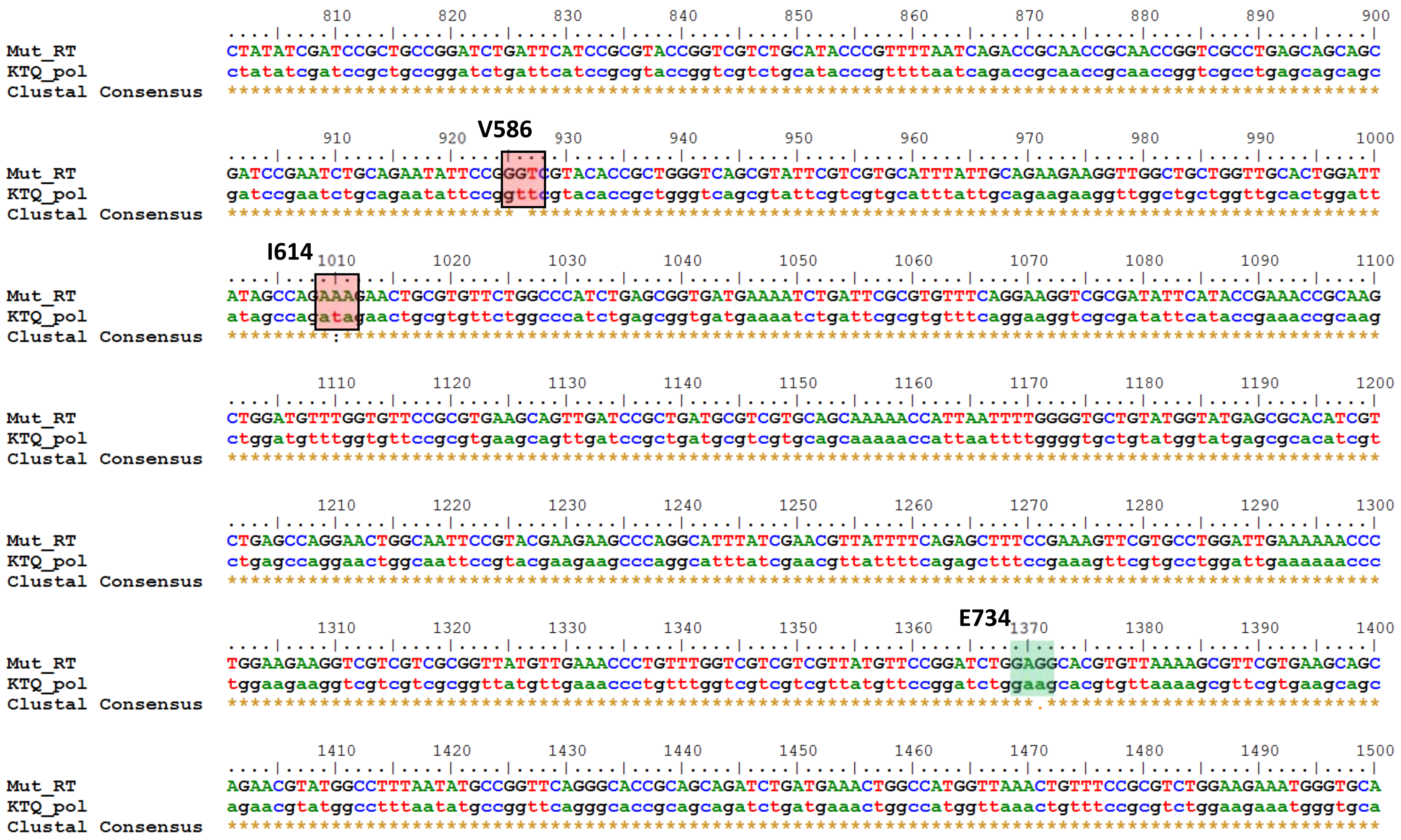

Mut_RT
KTQ_pol
Clustal Consensus
V586
I614
E734

```
                     1510      1520      1530      1540      1550      1560      1570      1580      1590      1600
                 ....|....|....|....|....|....|....|....|....|....|....|....|....|....|....|....|....|....|....|....|
Mut_RT             CGTATGCTGCTGCAGGTTCATGATGAACTGGTGCTGGAAGCACCGAAAGAACGTGCAGAAGCAGTTGCCCGTCTGGCAAAAGAAGTTATGGAAGGCGTTT
KTQ_pol            cgtatgctgctgcaggttcatgatgaactggtgctggaagcaccgaaagaacgtgcagaagcagttgcccgtctggcaaaagaagttatggaaggcgttt
Clustal Consensus  ****************************************************************************************************

                     1610      1620      1630      1640      1650      1660
                 ....|....|....|....|....|....|....|....|....|....|....|....|....|...
Mut_RT             ATCCGCTGGCAGTTCCGCTGGAAGTTGAAGTTGGTATTGGTGAAGATTGGCTGTCTGCaAAAGaAtaa
KTQ_pol            atccgctggcagttccgctggaagttgaagttggtattggtgaagattggctgtctgcaaaagaa---
Clustal Consensus  *****************************************************************
```

Figure S9.

Nucleotide sequence alignment of wild type KlenTaq DNA polymerase and Mut_RT. Red boxes with solid black line marks the target sites of mutations and the change in codon. The numbering in alignment ruler are arbitary. Position E734 marked in green box without border line, was not a target site for mutagenesis and a silent mutation (A→G) had occurred during PCR amplifcation.

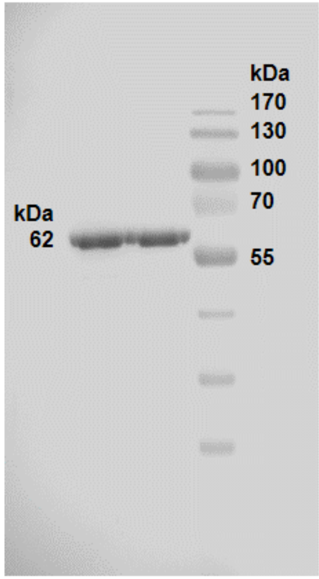

Figure S10 .

SDS-PAGE of Ni-NTA purified 6X-His tagged WT (left) and Mut_RT (middle).

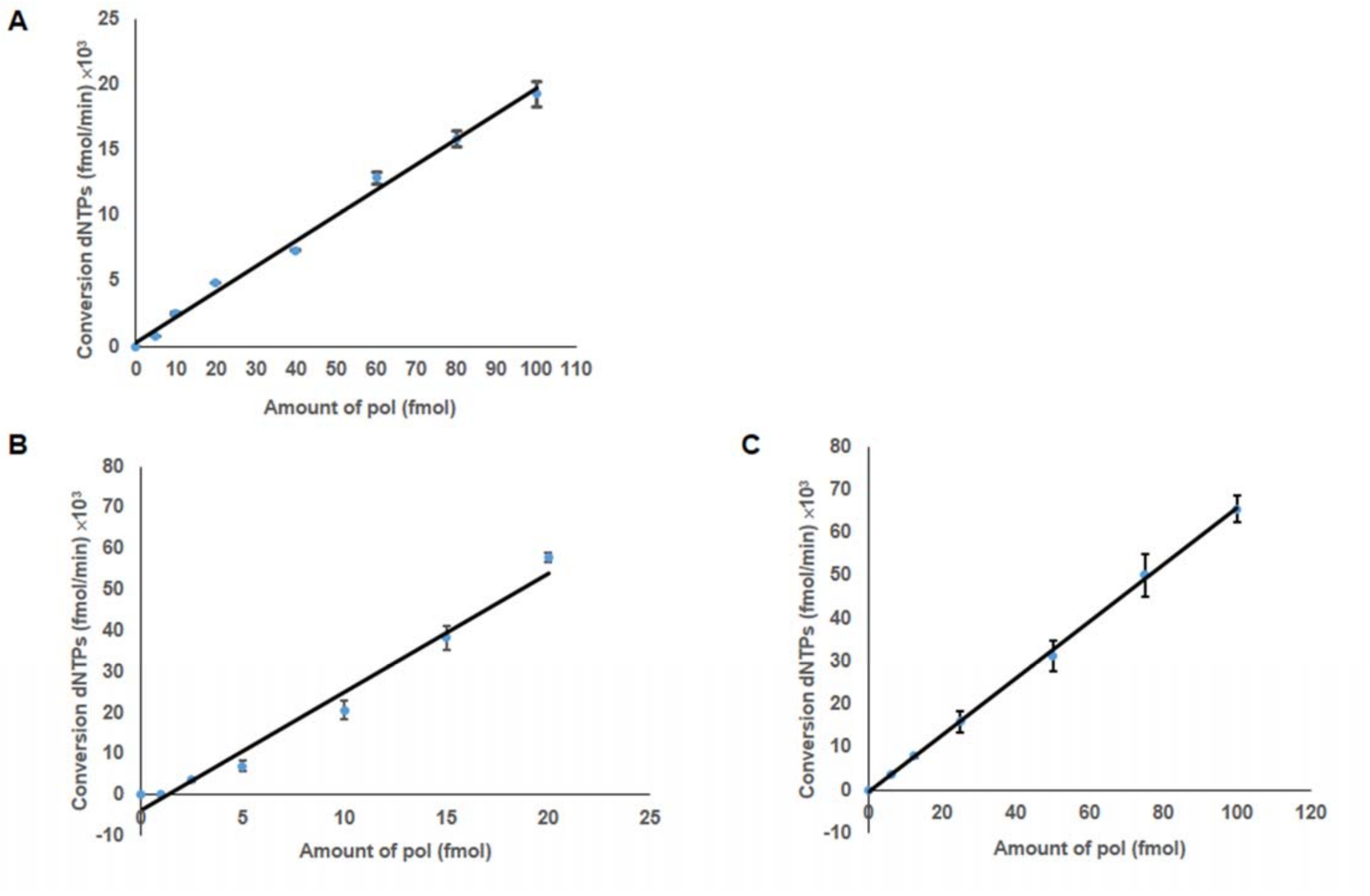

Figure S11.

Specific activities. (A) dNTP conversion rate plotted against the amount of Mut_RT applied on RNA template. dNTP conversion rate of Mut_RT (B) and wild type KlenTaq DNA polymerase (C) plotted against the amount of enzyme applied on DNA template. Error bars describe SD (n = 3).

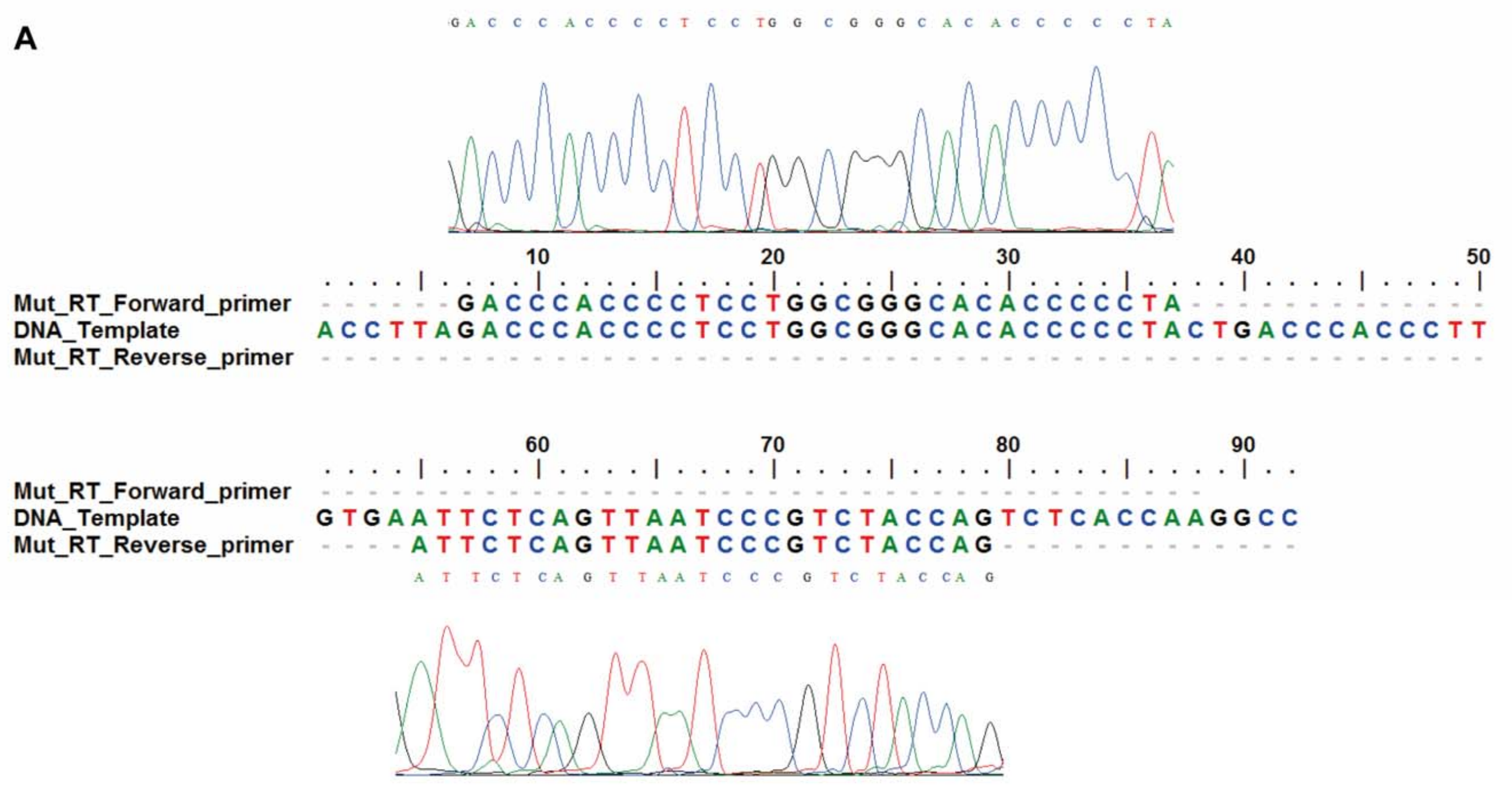
A
GACCCACCCCTCCTGGCGGGCACACCCCCTA
10 20 30 40 50
Mut_RT_Forward_primer GACCCACCCCTCCTGGCGGGCACACCCCCTA
DNA_Template ACCTTAGACCCACCCCTCCTGGCGGGCACACCCCCTACTGACCCACCCTT
Mut_RT_Reverse_primer
60 70 80 90
Mut_RT_Forward_primer
DNA_Template GTGAATTCTCAGTTAATCCCGTCTACCAGTCTCACCAAGGCC
Mut_RT_Reverse_primer ATTCTCAGTTAATCCCGTCTACCAG
ATTCTCAGTTAATCCCGTCTACCAG

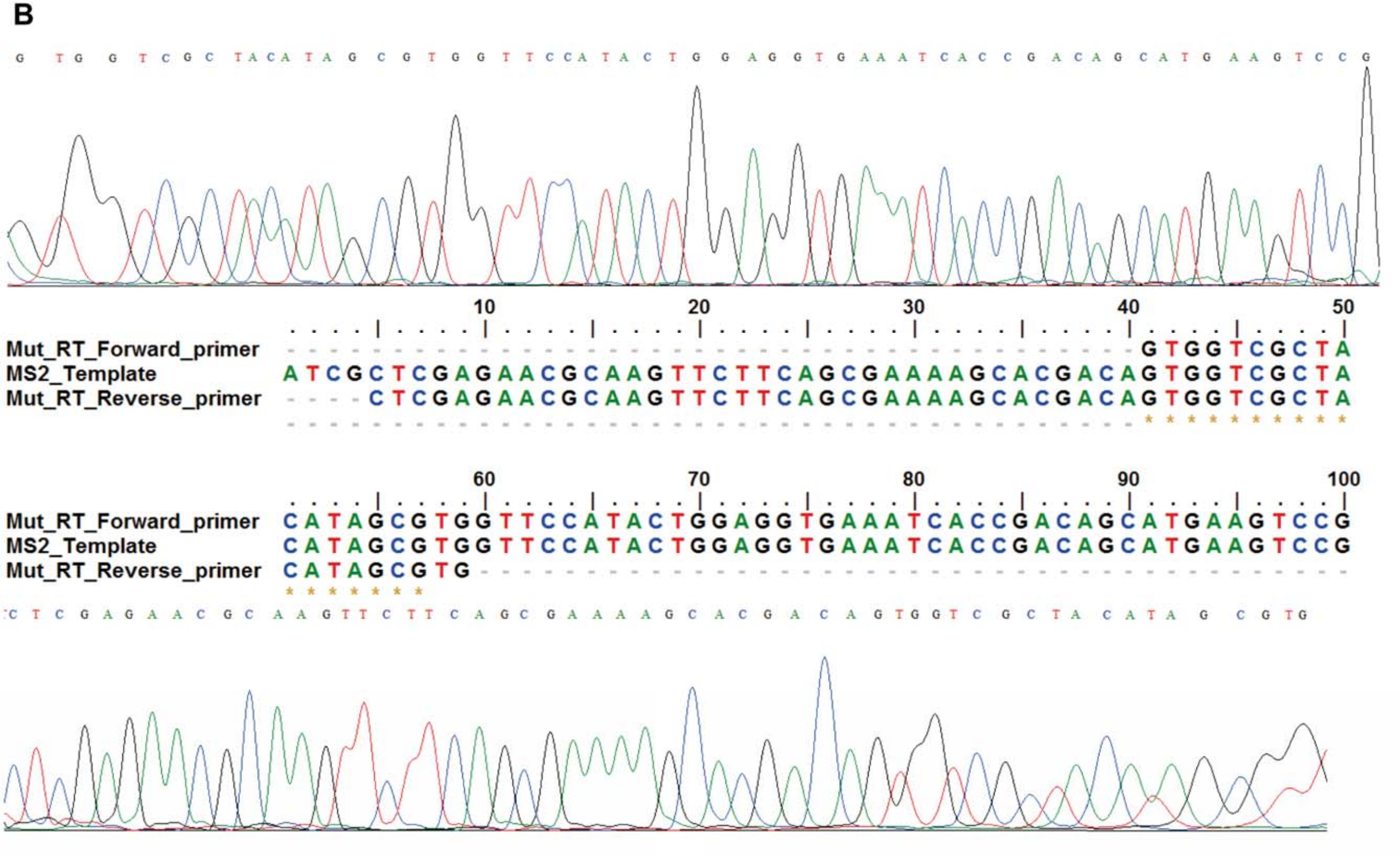

Figure S12. Sanger sequencing analysis of Mut_RT amplified products. A) Amplificate from DNA dependent DNA synthesis was presented in sequence alignment. Section of electropherogram of sequencing reactions with forward primer [5'-d(CTT GGT GAG ACT GGT AGA CG)-3'] (above sequence alignment) and reverse primer [5'-d(TTA GAC CCA CCC CTC CTG GCG)-3'] (below sequence alignment) respectively were shown. B) Amplificate from RNA dependent DNA synthesis. Sequenced with primers RT_For (forward primer) and RT_Rev (Reverse primer) of MS2 template.

Figure S13 (Full image of 4A)

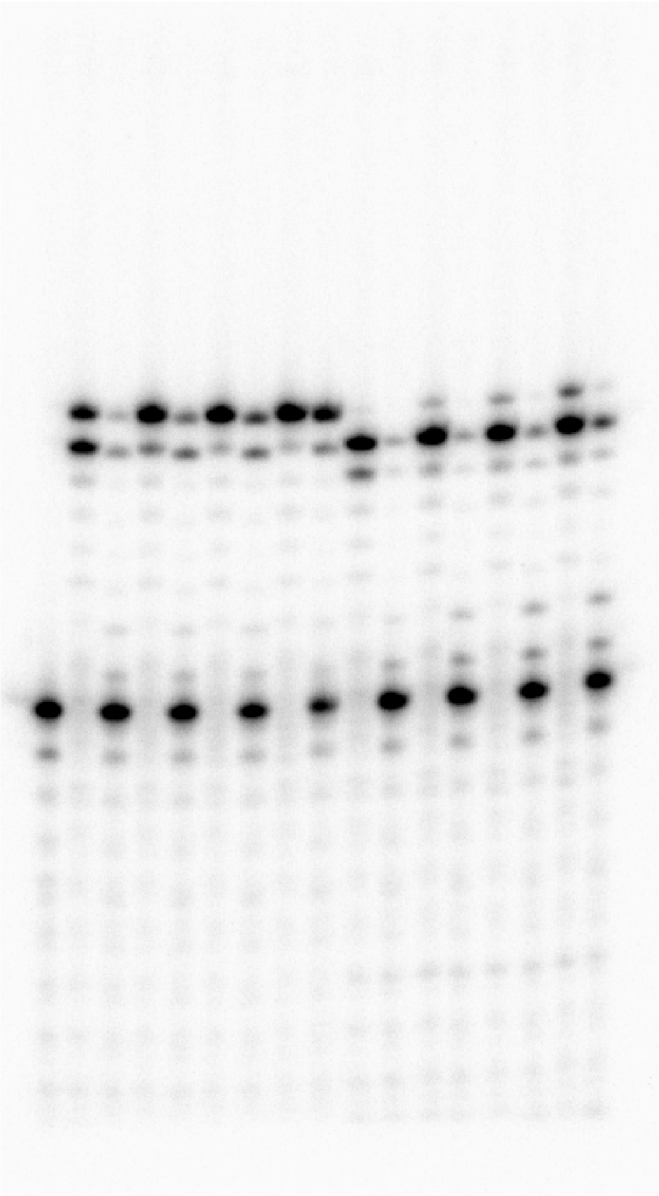

Figure S14 (Full image of 5C)

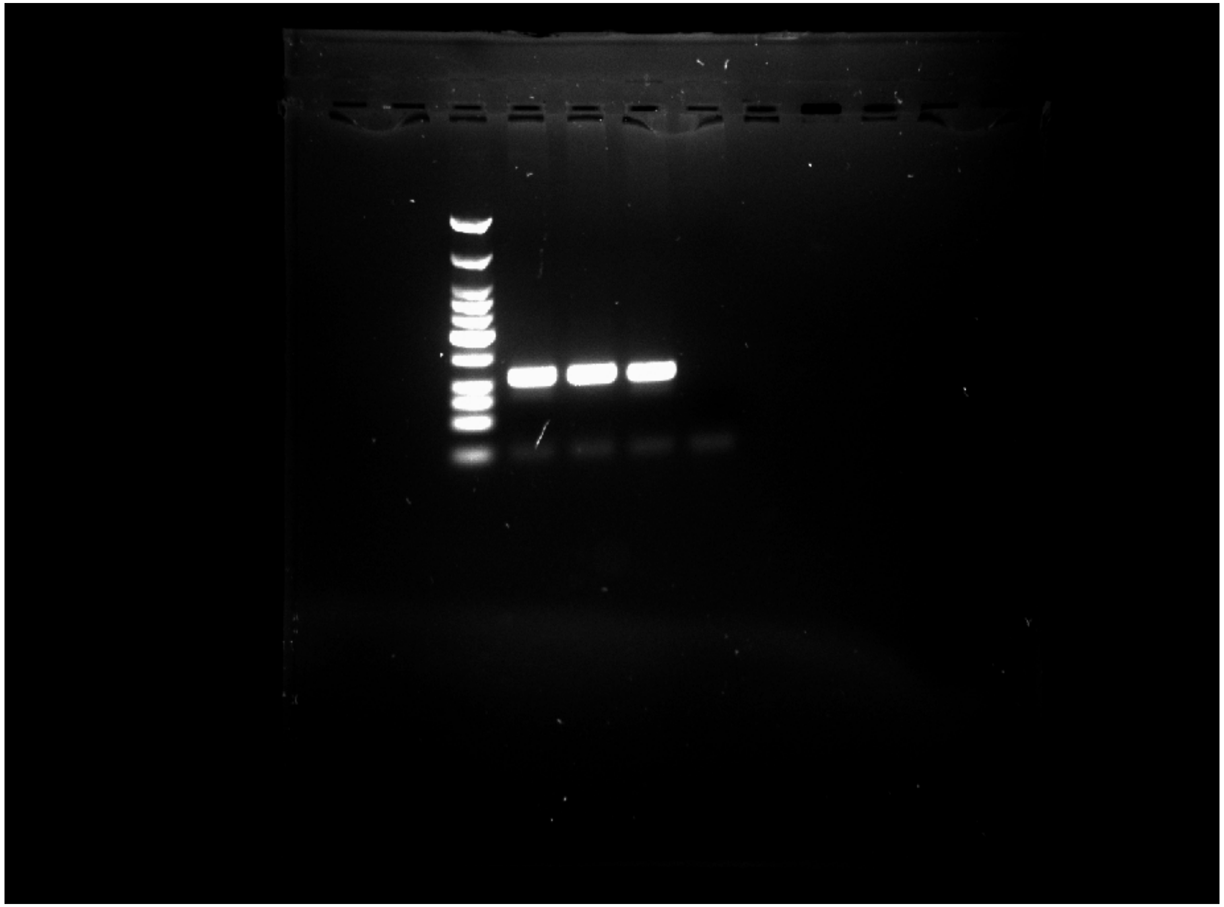

Figure S15 (Full image of 5E)

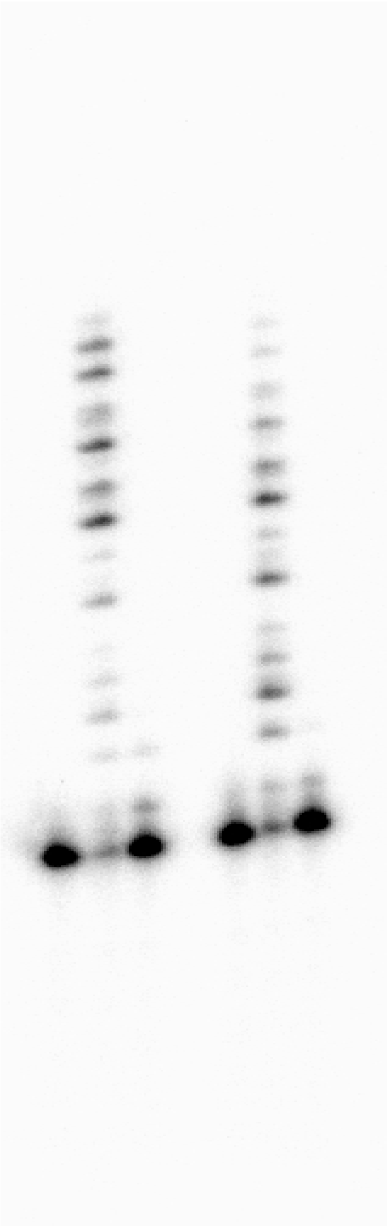

Figure S16 (Full image of 6B)

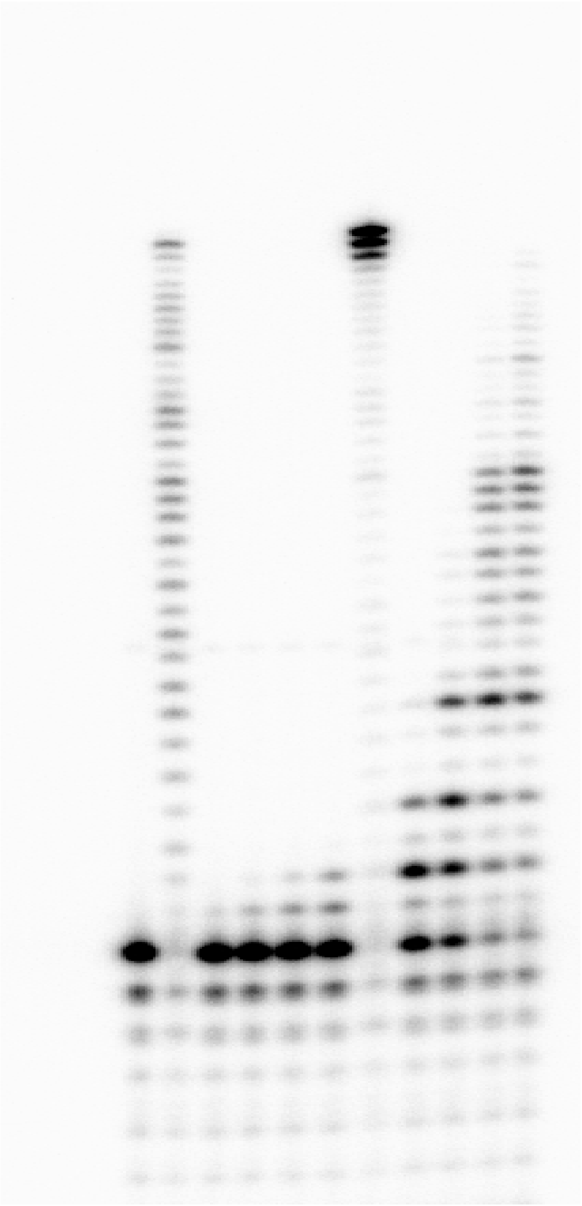

Figure S17 (Full image of 6D)

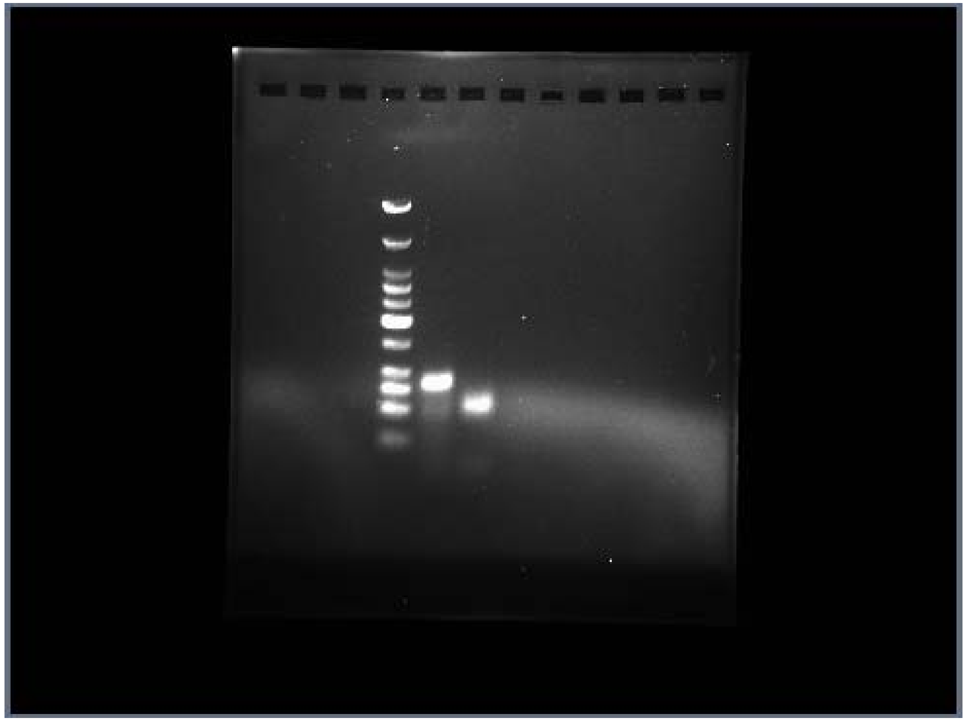

Figure S18 (Full image of S7)

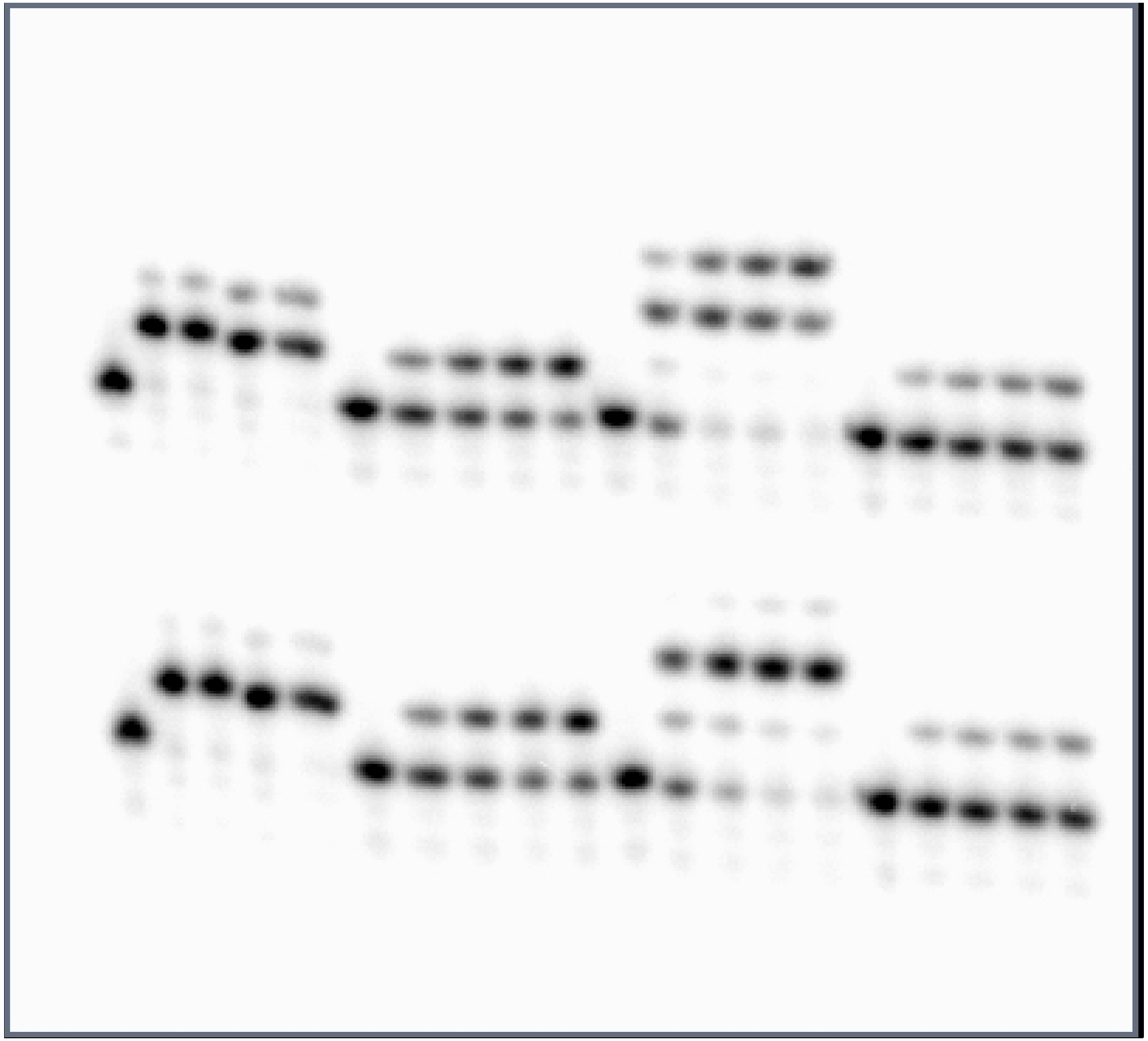

Figure S19 (Full image of S8)

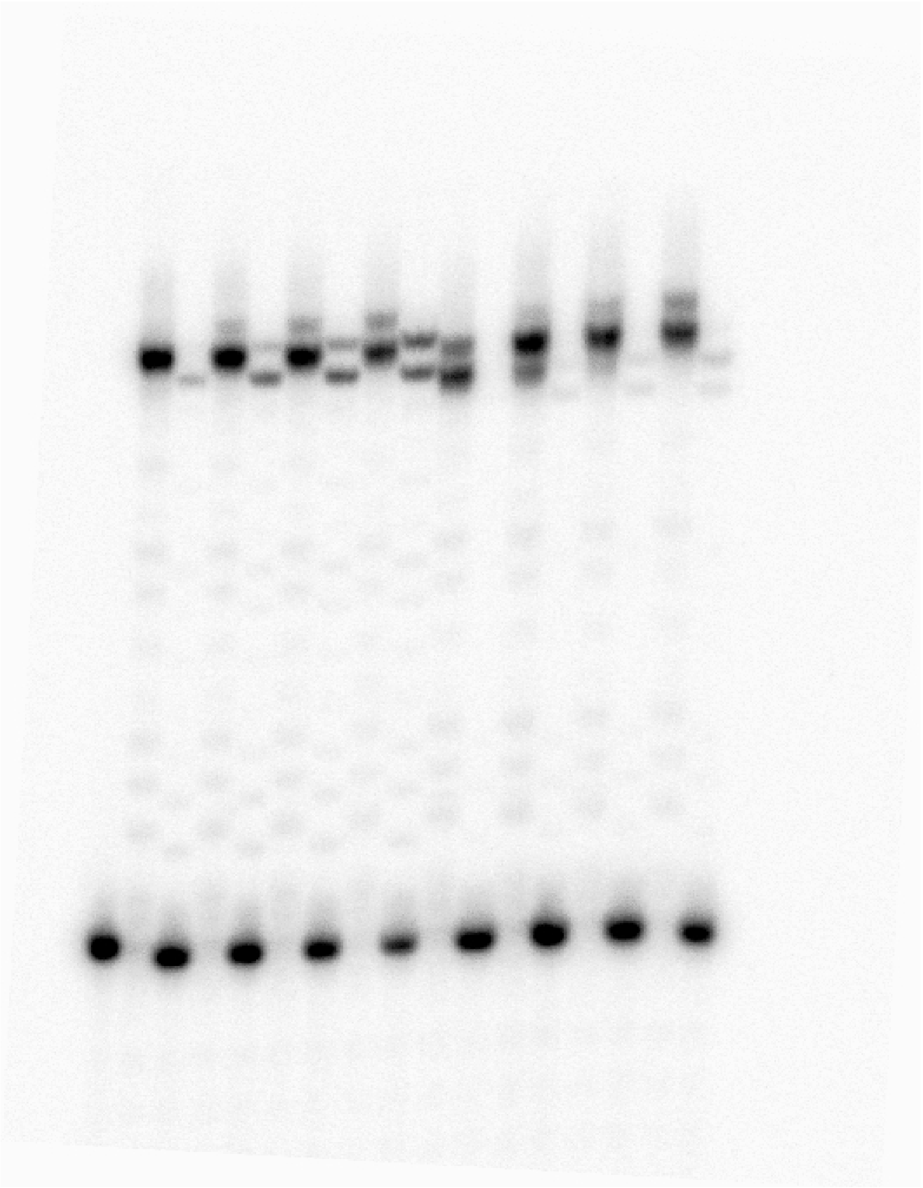

Reference:

1. Li,J., Han,C., Deng,J. and Wang,H. Cloning and Preliminary Characterization of Human Nanog Promoter, Unpublished, GenBank: GU433186.1 GI: 289547169, Animal Biotechnology, Northwest A&F University, Taicheng Road No. 3, Yangling, Shaanxi 712100, China
